# The naked truth: a comprehensive clarification and classification of current ‘myths’ in naked mole‐rat biology

**DOI:** 10.1111/brv.12791

**Published:** 2021-09-03

**Authors:** Rochelle Buffenstein, Vincent Amoroso, Blazej Andziak, Stanislav Avdieiev, Jorge Azpurua, Alison J. Barker, Nigel C. Bennett, Miguel A. Brieño‐Enríquez, Gary N. Bronner, Clive Coen, Martha A. Delaney, Christine M. Dengler‐Crish, Yael H. Edrey, Chris G. Faulkes, Daniel Frankel, Gerard Friedlander, Patrick A. Gibney, Vera Gorbunova, Christopher Hine, Melissa M. Holmes, Jennifer U. M. Jarvis, Yoshimi Kawamura, Nobuyuki Kutsukake, Cynthia Kenyon, Walid T. Khaled, Takefumi Kikusui, Joseph Kissil, Samantha Lagestee, John Larson, Amanda Lauer, Leonid A. Lavrenchenko, Angela Lee, Jonathan B. Levitt, Gary R. Lewin, Kaitlyn N. Lewis Hardell, TzuHua D. Lin, Matthew J. Mason, Dan McCloskey, Mary McMahon, Kyoko Miura, Kazutaka Mogi, Vikram Narayan, Timothy P. O'Connor, Kazuo Okanoya, M. Justin O'Riain, Thomas J. Park, Ned J. Place, Katie Podshivalova, Matthew E. Pamenter, Sonja J. Pyott, Jane Reznick, J. Graham Ruby, Adam B. Salmon, Joseph Santos‐Sacchi, Diana K. Sarko, Andrei Seluanov, Alyssa Shepard, Megan Smith, Kenneth B. Storey, Xiao Tian, Emily N. Vice, Mélanie Viltard, Akiyuki Watarai, Ewa Wywial, Masanori Yamakawa, Elena D. Zemlemerova, Michael Zions, Ewan St. John Smith

**Affiliations:** ^1^ Calico Life Sciences LLC 1170 Veterans Blvd South San Francisco CA 94080 U.S.A.; ^2^ Department of Biological Sciences University of Illinois at Chicago Chicago IL 60607 U.S.A.; ^3^ Graduate Center City University of New York 365 Fifth Avenue New York NY 10016 U.S.A.; ^4^ Moffitt Cancer Center 12902 Magnolia Drive Tampa FL 33612 U.S.A.; ^5^ Department of Anesthesiology Stony Brook University 101 Nicolls Road Stony Brook NY 11794 U.S.A.; ^6^ Max Delbrück Center for Molecular Medicine Robert‐Rössle‐Str 10 Berlin‐Buch 13092 Germany; ^7^ Mammal Research Institute, Department of Zoology and Entomology University of Pretoria Pretoria 0002 South Africa; ^8^ Department of Obstetrics, Gynecology & Reproductive Medicine Magee‐Womens Research Institute 204 Craft Avenue Pittsburgh PA 15213 U.S.A.; ^9^ Department Biological Sciences Rondebosch Cape Town 7701 South Africa; ^10^ Reproductive Neurobiology, Division of Women's Health School of Medicine, King's College London Westminster Bridge Road London SE1 7EH U.K.; ^11^ Zoological Pathology Program University of Illinois 3505 Veterinary Medicine Basic Sciences Building, 2001 S Lincoln Avenue Urbana IL 6180 U.S.A.; ^12^ Department of Pharmaceutical Sciences Northeast Ohio Medical University 4209 State Route 44 Rootstown OH 44272 U.S.A.; ^13^ Northwest Vista College 3535 N. Ellison Drive San Antonio TX 78251 U.S.A.; ^14^ School of Biological and Chemical Sciences Queen Mary University of London Mile End Road London E1 4NS U.K.; ^15^ School of Engineering Newcastle University Merz Court Newcastle Upon Tyne NE1 7RU U.K.; ^16^ Université Paris Descartes Faculté de Médecine 12 Rue de l'École de Médecine Paris 5006 France; ^17^ Cornell University College of Veterinary Medicine Ithaca NY 14853 U.S.A.; ^18^ Departments of Biology University of Rochester 402 Hutchison Hall Rochester NY 14627 U.S.A.; ^19^ Cleveland Clinic Lerner Research Institute 9500 Euclid Avenue Cleveland OH 44195 U.S.A.; ^20^ Department of Psychology University of Toronto Mississauga 3359 Mississauga Road North Mississauga ON L5L 1C6 Canada; ^21^ Department of Aging and Longevity Research Kumamoto University 1‐1‐1 Honjo Kumamoto 860‐0811 Japan; ^22^ Department of Evolutionary Studies of Biosystems The Graduate University for Advanced Studies Hayama 240‐0193 Japan; ^23^ The School of the Biological Sciences University of Cambridge Tennis Court Road Cambridge CB2 1PD U.K.; ^24^ Companion Animal Research, School of Veterinary Medicine Azabu University Sagamihara 252‐5201 Japan; ^25^ Department of Cancer Biology The Scripps Research Institute Scripps Florida Jupiter FL 33458 U.S.A.; ^26^ Department of Otolaryngology Johns Hopkins School of Medicine Baltimore MD 21205 U.S.A.; ^27^ A.N. Severtsov Institute of Ecology and Evolution Russian Academy of Sciences Leninskii pr. 33 Moscow 119071 Russia; ^28^ Biology Department The City College of New York 138th Street and Convent Avenue New York NY 10031 U.S.A.; ^29^ College of Staten Island in the City University of New York 2800 Victory Blvd Staten Island NY 10314 U.S.A.; ^30^ The Rockefeller University 1230 York Avenue New York NY 10065 U.S.A.; ^31^ Department of Life Sciences The University of Tokyo 7‐3‐1 Hongo Tokyo 153‐8902 Japan; ^32^ Biology Department University of Ottawa 30 Marie Curie Ottawa ON K1N 6N5 Canada; ^33^ Groningen Department of Otorhinolaryngology University Medical Center Postbus 30.001 Groningen RB 9700 The Netherlands; ^34^ Cologne Excellence Cluster for Cellular Stress Responses in Aging‐Associated Diseases (CECAD) University Hospital Cologne Joseph‐Stelzmann‐Street 26 Cologne 50931 Germany; ^35^ Barshop Institute for Longevity and Aging Studies University of Texas Health Science Center 4939 Charles Katz Dr. San Antonio TX 78229 U.S.A.; ^36^ Department of Neuroscience Yale University School of Medicine 200 South Frontage Road, SHM C‐303 New Haven CT 06510 U.S.A.; ^37^ Department of Anatomy School of Medicine, Southern Illinois University 975 S. Normal Carbondale IL 62901 U.S.A.; ^38^ Department of Biology Carleton University 1125 Colonel By Drive Ottawa ON K1S 5B6 Canada; ^39^ Department of Genetics – Blavatnik Institute Harvard Medical School 77 Avenue Louis Pasteur Boston MA 02115 U.S.A.; ^40^ Fondation pour la recherche en Physiologie Université Catholique de Louvain Clos Chapelle‐aux‐Champs 30 Woluwe‐saint Lambert 1200 Belgium

**Keywords:** naked mole‐rat, longevity, hypoxia, cancer, nociception, eusociality, cancer, thermoregulation, ageing, ecology

## Abstract

The naked mole‐rat (*Heterocephalus glaber*) has fascinated zoologists for at least half a century. It has also generated considerable biomedical interest not only because of its extraordinary longevity, but also because of unusual protective features (e.g. its tolerance of variable oxygen availability), which may be pertinent to several human disease states, including ischemia/reperfusion injury and neurodegeneration. A recent article entitled ‘Surprisingly long survival of premature conclusions about naked mole‐rat biology’ described 28 ‘myths’ which, those authors claimed, are a ‘perpetuation of beautiful, but falsified, hypotheses’ and impede our understanding of this enigmatic mammal. Here, we re‐examine each of these ‘myths’ based on evidence published in the scientific literature. Following Braude *et al*., we argue that these ‘myths’ fall into four main categories: (*i*) ‘myths’ that would be better described as oversimplifications, some of which persist solely in the popular press; (*ii*) ‘myths’ that are based on incomplete understanding, where more evidence is clearly needed; (*iii*) ‘myths’ where the accumulation of evidence over the years has led to a revision in interpretation, but where there is no significant disagreement among scientists currently working in the field; ( *iv* ) ‘myths’ where there is a genuine difference in opinion among active researchers, based on alternative interpretations of the available evidence. The term ‘myth’ is particularly inappropriate when applied to competing, evidence‐based hypotheses, which form part of the normal evolution of scientific knowledge. Here, we provide a comprehensive critical review of naked mole‐rat biology and attempt to clarify some of these misconceptions.

## I. INTRODUCTION

Although naked mole‐rats (*Heterocephalus glaber*) were first described more than 150 years ago (Rüppell, 1842) it is only over the last 40 years that scientists have begun to dig deeply into the many facets of their unusual biology (Buffenstein, Park & Holmes, 2021). The naked mole‐rat has not only garnered attention for being a eusocial mammal (Jarvis, 1981; Holmes & Goldman, 2021), highly specialised for life underground through sensory (Lewin *et al*., 2021; Vice *et al*., 2021) and ecophysiological adaptations (Buffenstein & Craft, 2021), but it has also been used in biomedical studies. These include research on pain (Park *et al*., 2008; Eigenbrod *et al*., 2019) hypoxia tolerance (Larson *et al*., 2014; Park *et al*., 2017, 2021; Ivy *et al*., 2020), cardiac function (Grimes *et al*., 2014*b*), cancer (Tian *et al*., 2013; Miyawaki *et al*., 2016; Taylor, Milone & Rodriguez, 2017; Seluanov *et al*., 2018; Shepard & Kissil, 2020; Delaney, Imai & Buffenstein, 2021), neuropeptidergic control of reproduction and behaviour (Coen *et al*., 2021), and ageing (Buffenstein, 2005; Lewis & Buffenstein, 2016; Ruby, Smith & Buffenstein, 2018). Collectively, these studies constitute a new paradigm recognising the naked mole‐rat as an important non‐traditional biomedical model for understanding how evolutionary processes optimise physiological mechanisms for survival under adverse conditions encountered in challenging habitats. Investigating these evolved systems in eusocial naked mole‐rats, which are adapted to crowded, hot, arid, and resource‐restricted niches in East Africa, also allows us to understand better mechanistic aspects of several human diseases that share features in common with subterranean ecophysiological stressors (Buffenstein & Ruby, 2021).

A recent review of naked mole‐rat biology (Braude *et al*., 2021) – referred to as ‘Braude *et al*.’ herein – identified 28 ‘myths’ (see Table 1) relating to naked mole‐rats, and concluded that many are a ‘perpetuation of beautiful, but falsified, hypotheses’, or speculation ‘perpetuated as fact’ (Braude *et al*., 2021, p. 377). In effect, they challenge numerous aspects of the naked mole‐rat paradigm.

**Table 1:** A summary of the 28 ostensible ‘myths’ of Braude *et al*. (2021) regarding naked mole‐rat biology and our categorisation based upon available evidence. The origin of each myth is classified as (1) myths that are in headline form and hence represent oversimplifications or are of unknown provenance; (2) suppositions requiring more evidence; (3) former conclusions that have been revised in the scientific literature; and (4) differing interpretations of available evidence

Myth	Response	1	2	3	4
Myth 1: naked mole‐rats are hairless	Naked mole‐rats do have a few hairs on their body, but they lack a fur coat. The sparse hairs are specialised sensory structures, similar to facial vibrissae. These hairs are nevertheless fundamentally different from pelage (hair or fur).	x			
Myth 2: naked mole‐rats are strictly subterranean and never go above ground	Naked mole‐rats have been found above ground, although this is a very rare occurrence, and the cause and purpose of these rare events is unknown. There is no clear evidence that they forage, find mates, or disperse above ground. Rather, their rare appearances on the surface may be due to very rare dispersals or colony displacement from disturbed, breached, or flooded burrows.	x	x		x
Myth 3: naked mole‐rats have unusually long burrows	Irrespective of whether naked mole‐rats in future studies remain the record holder for the longest burrow system among the social mole‐rats, naked mole‐rats have exceptionally long burrows for their body size and colony biomass.	x			
Myth 4: naked mole‐rats are the only poikilothermic mammals	This myth is predicated on two words – ‘only’ and ‘poikilothermic’. Some other mole‐rat species also show pronounced thermolability. Body temperature (*T*_b_) of naked mole‐rats, when given sufficient time to attain thermal equilibrium, closely tracks that of ambient temperature (*T*_a_), being only slightly warmer than *T*_a_. They nevertheless can and do employ endothermy, however this is ineffective at maintaining body temperature outside of a very narrow range of *T*_a_.	x			x
Myth 5: naked mole‐rats have uniquely low thyroid hormone levels	‘Unique’ is a perilous word, but naked mole‐rats certainly have low free thyroxine (T4) levels. However, to date, neither triiodothyronine (T3) level nor the T4:T3 ratio have been reported in the peer‐reviewed literature.	x	x		
Myth 6: naked mole‐rat burrows are hypoxic and hypercapnic	Measuring underground atmospheres without altering their composition is difficult. To date, oxygen and carbon dioxide levels have not been measured in nest chambers. Burrow composition, organisation, and animal density would suggest that naked mole‐rats encounter a range of atmospheric conditions, including hypoxia and hypercapnia, in their daily routine. Also, numerous physiological traits demonstrate that naked mole‐rats are extremely well‐adapted to both hypoxic and hypercapnic conditions.	x	x		
Myth 7: naked mole‐rats are blind	The naked mole‐rat visual system is degenerate, with significant atrophy of the structures required for visual image formation. Their visual cortex is primarily responsive to somatosensory inputs. However, some ancillary structures remain intact, and while they can detect changes in luminance, they cannot see images.	x			
Myth 8: naked mole‐rats have degenerated hearing	Compared with surface‐dwelling mammals, naked mole‐rats have very restricted high‐frequency hearing, high auditory thresholds, and very poor sound‐localisation abilities. Questions remain about how their low‐frequency hearing compares with that of related species.	x			x
Myth 9: naked mole‐rats are the most vocal rodents because they live in large groups	Current evidence does not permit group size to be discounted as an important contributor to vocal complexity; it is likely to be one of several key evolutionary drivers that shaped the extensive range of the naked mole‐rat's vocal repertoire.		x		
Myth 10: naked mole‐rats feel no pain	This claim has no basis in the scientific literature. Naked mole‐rats do respond to certain noxious stimuli (e.g. heat and mustard oil), but lack responses to others, such as acid and capsaicin.	x			
Myth 11: naked mole‐rats are the only eusocial mammals	The definition of the term ‘eusocial’ is controversial and has been extensively debated. Both naked and Damaraland mole‐rats appear distinct from other species in that they have very high levels of skew in lifetime reproductive success, but solid evidence for task specialisation among workers is lacking.	x	x	x	x
Myth 12: colonies have castes of breeders and non‐breeders, involving frequent workers, infrequent workers, non‐workers, and dispersers	There is no disagreement about distinct reproductive division of labour, accompanied by some morphological specialisation in breeding queen naked mole‐rats. However, there is no conclusive evidence of distinct worker and non‐worker castes among non‐breeding animals.	x	x	x	x
Myth 13: colonies have up to three male breeders (pashas)	Whilst copulation does not guarantee paternity, up to three male consorts have been reported, with evidence from both behavioural and genetic studies. It is not known if there could be more than three breeding males within a colony.	x	x		
Myth 14: colonies have a single queen	Dual queening is well documented, but it is a very rare event both in captivity and in the wild. To date, there has been no recorded incidence of three or more reproductive females within a colony, and most colonies only have a single queen.	x			
Myth 15: not all females can become queens	When isolated from a colony, most new pairs will breed. However, not all females appear capable of attaining and holding the dominant position of queen within a colony.	x	x		
Myth 16: queens suppress workers with pheromones	Initial suggestion that pheromones ‘may’ be involved in suppression was revised in the 1990s as behavioural contact between queen and non‐breeders was recognised as key to reproductive suppression. To date, there is no conclusive evidence to suggest a role for pheromones in reproductive suppression.	x		x	
Myth 17: queens shove workers to get them to work	Since the original paper proposing this, this idea has been debunked in multiple studies.			x	
Myth 18: naked mole‐rats never leave their natal colonies	Genetic evidence supports outbreeding as vital to the evolutionary ecology of the naked mole‐rat. Some dispersal from the natal nest is thus required.	x	x		
Myth 19: naked mole‐rats are inbred	While facultative inbreeding does occur in captivity, it is rare in other social mole‐rats. While early studies in the 1980s of a single population suggested extreme inbreeding, more recent studies reveal considerable heterozygosity in both wild and captive populations.			x	
Myth 20: the GH/IGF axis is impaired in naked mole‐rats	Substantial data support the premise that the GH/IGF axis is indeed dampened in this species.	x	x		x
Myth 21: naked mole‐rats are long‐lived because they have low oxidative stress and damage	Although naked mole‐rats have high levels of oxidative tissue damage, this manifests early in captive life and does not accumulate with age. This suggests that naked mole‐rats can efficiently neutralise or repair oxidative damage. Production of reactive oxygen species and antioxidant expression vary among tissues.	x	x	x	
Myth 22: naked mole‐rat cells do not display cellular senescence	While equivocal findings have been reported, it appears that naked mole‐rat cells can undergo stress and oncogene‐induced senescence *in vitro*.		x		
Myth 23: naked mole‐rats are immune to disease	Compared to other rodents in managed care, naked mole‐rats have fewer documented diseases including age‐related dysfunction (e.g. neurodegeneration, heart and kidney disease) and bacterial infections. Rare, lethal viral infections have been reported.	x	x		
Myth 24: naked mole‐rats do not get tumours or cancer	Although several cases of cancer have been identified in this species, the incidence of spontaneous neoplasia in naked mole‐rats relative to other similarly sized mammals and even humans is extremely low.	x			
Myth 25: naked mole‐rats have extremely large hyaluronan	Naked mole‐rat hyaluronan is larger than hyaluronan from several other mammals examined and has unusual material properties. Results may differ among research groups due to hyaluronan molecular weight being affected by the isolation procedure.		x	x	x
Myth 26: naked mole‐rat cells have early contact inhibition that prevents cancer	Naked mole‐rat cell growth is highly dependent upon tissue culture conditions, which can impede cell proliferation prior to confluence and exhibit contact inhibition properties. Under certain conditions (e.g. daily medium changes), cells grow to high density and show no signs of contact inhibition.	x		x	
Myth 27: naked mole‐rats are non‐aging	Demographic data show no increased mortality risk as animals increase in age. Also, many physiological phenotypes show negligible age‐related changes. These data support the premise of a non‐ageing mammal and/or suggest that morbidities are likely compressed into the final years of life.	x	x		
Myth 28: naked mole‐rats are the single member of a taxonomic family	Whether the naked mole‐rat is a single member of the family Heterocephalidae or a member of the family Bathyergidae that also includes other sub‐Saharan mole‐rats is controversial. The Sub‐Saharan African mole‐rats nevertheless appear to be a monophyletic group regardless of whether they are considered a single family or a superfamily.		x		x

In response, we here re‐examine each of these ‘myths’. We provide a comprehensive critical review and clarifying compilation of our current knowledge about naked mole‐rat biology. We re‐examine the context of evidence and illuminate certain experimental details that we believe may have been misinterpreted. We argue that many of the so‐called ‘myths’ are in fact supported by the available evidence, while others are based on over‐simplifications. As correctly pointed out by Braude *et al*., some incorrect ideas may indeed be propagated in the popular press, although this is generally not the case in the scientific literature. Nascent hypotheses are continuously refined as new data are acquired; such is the nature of the ‘re‐’ in ‘research’. There are also ongoing differences in interpretation of data, which are inevitable in an emerging but rapidly growing field. We will demonstrate that, although more work is certainly needed in many areas, we have a much more solid understanding of naked mole‐rat biology than implied by Braude *et al*. (2021).

## II. ECOPHYSIOLOGY AND ENVIRONMENT

### (1) Myth 1: naked mole‐rats are hairless

Both the common name and the scientific name of the naked mole‐rat (*H. glaber*) reflect the fact that these animals, unlike most other mammals, lack an insulatory pelage made of fine, densely packed hairs (Cui *et al*., 2020). As such, naked mole‐rats are ‘naked’ in much the same way as Desmond Morris saw us, in his book *The Naked Ape* (Morris, 1967).

**Fig 1:**
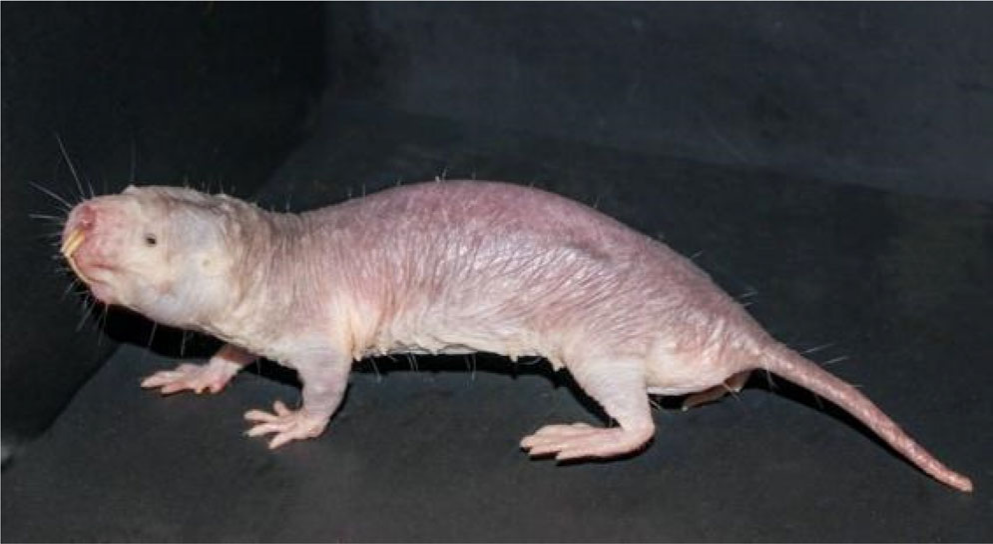
Sparsely distributed sensory vibrissae, although distributed all over the body, are very different to the fine, dense hair that constitutes the fur of most mammals. Photograph credit: Megan Smith.

Braude *et al*. are correct in stating that naked mole‐rats are not strictly hairless. They possess carefully arranged grids of sparse, whisker‐like guard hairs or vibrissae that criss‐cross their body or surround their mouth and ears (Fig. 1). These vibrissae do not have a thermoregulatory role, but rather are specialised, highly innervated sensory tactile organs (Crish *et al*., 2003). Indeed, Lewin *et al*. (2021) made direct recordings from sensory afferents innervating these hairs and showed that they have the unusual property of signalling the direction of hair deflection. Sensory afferents innervating these hairs could provide the animal with information about the direction and speed of their movement in narrow tunnels (Lewin *et al*., 2021). Braude *et al*. refer to two papers loosely describing naked mole‐rats as hairless, but such claims are certainly not widespread in the scientific literature (Daly & Buffenstein, 1998; Crish *et al*., 2003; Park *et al*., 2003; Lewin *et al*., 2021).

### (2) Myth 2: naked mole‐rats are strictly subterranean and never go above ground

Naked mole‐rats have been found in pit‐traps or wandering on the surface during fieldwork, revealing that they do occasionally go above ground. As such, we agree that the statement in the subtitle above is untrue. Braude *et al*. go on to conclude that ‘while there is no direct evidence of above‐ground foraging, naked mole‐rats do disperse above ground’ (Braude *et al*., 2021, p. 378). Biological dispersal refers to the movement of individuals from their birth or breeding site to another breeding site. Rare instances of above‐ground dispersal were originally proposed by O'Riain, Jarvis & Faulkes (1996) following the discovery of dispersive morphs in captive colonies. However, given their poor eyesight, limited thermoregulatory abilities, very fine, permeable skin, and their inability to jump or hop out of reach of a predator, it is unlikely that these rodents would survive for long above ground in the arid conditions of East Africa. Wandering on the surface to find a mate, pairing, and thereafter excavating a new burrow system, or going in search of a neighbouring open burrow, would be a highly risky and life‐threatening strategy. Braude *et al*. themselves report that they caught only nine mole‐rats in pit‐traps over the duration of their three‐year study. This supports the premise that this is a very rare occurrence (Braude *et al*., 2020), and may possibly have been due to burrow damage during fieldwork. If animals indeed venture above ground for the purpose of dispersal, this has not yet been proved.

In order better to understand why naked mole‐rats might occasionally be found on the surface, we can look at other bathyergids. In over 10 years of fieldwork during both summer and winter seasons, neither Damaraland (*Fukomys damarensis*) nor common (*Cryptomys hottentotus*) mole‐rats were ever found above ground (N.C. Bennett & J.U.M. Jarvis, personal observations). However, one study reported that numerous Damaraland mole‐rats were found in a 2 m deep concrete‐lined water channel in Otjiwarongo, Namibia (Hazell *et al*., 2000). Closer inspection revealed that the concrete edges of the channel formed a barrier to burrowing, thus forcing mole‐rats onto the surface where they subsequently fell into the channel. The only mole‐rat that appears to be regularly found on the surface is the solitary Cape dune mole‐rat (*Bathyergus suillus*). It is typically found after the long winter rains in a hypothermic state, suggesting that burrow flooding was a causative factor (Bennett & Faulkes, 2000). We therefore believe that there is no strong evidence that either the naked mole‐rat or its larger, better‐insulated relatives commonly move above ground to disperse or forage.

### (3) Myth 3: naked mole‐rats have unusually long burrows

The social mole‐rats tend to live in burrow systems that may often span several hundred metres or more in total length (Bennett, 1988; Spinks, Bennett & Jarvis, 2000; Herbst & Bennett, 2006; Šklíba *et al*., 2012; Thomas *et al*., 2012; Thomas, Swanepoel & Bennett, 2016). Brett reports two naked mole‐rat burrows that were meticulously mapped having total tunnel lengths of 3027 m (Kamboyo 1) and 595 m (Lerata 1) (Brett, 1986, 1991). For Kamboyo 1, Brett gives an evidence‐based argument (see Brett, 1986) that this is an underestimate and that it was actually more likely to be 3.6–4.0 km. Even at the conservatively reported 3027 m, Kamboyo 1 is the largest mole‐rat burrow mapped to date, contradicting what has been claimed for *Fukomys mechowii*: ‘Both uncovered burrow systems were very large (total lengths, 2245 m and 743 m), making them the largest burrow systems ever mapped’ (Šumbera *et al*., 2012). Both studies used similar techniques involving radio‐tracking and excavation. Šumbera *et al*. (2012) compare the longer 2245 m *F. mechowii* burrow to the shorter ‘Lerata’ naked mole‐rat burrow of Brett's dissertation, but overlook the larger ‘Kamboyo 1’ burrow that was described in detail in the same paragraph (Brett, 1986). Furthermore, two of the *F. mechowii* burrows in Šumbera *et al*. (2012) were connected through a single blocked tunnel. It is difficult to ascertain if the excavated tunnel was created by one or more groups, potentially biasing those results. Braude *et al*. attempt to qualify the species comparison by saying that the *F. mechowii* burrow is the ‘largest burrow systems ever mapped in relation to biomass’ (Braude *et al*., 2021, p. 380), yet they do not provide the biomass value. Since Šumbera *et al*. (2012) reported that not all animals were caught and no breeding male was found, it is impossible to determine the biomass of that colony and so a direct comparison with naked mole‐rat biomass and burrow length cannot be made. More data are needed before comparisons of average tunnel length and colony biomass can be made between bathyergid species. Since *H. glaber* is less than half the body mass of *F. mechowii*, it would be true to say that both in absolute terms and relative to individual animal size, naked mole‐rats have the longest tunnel systems recorded to date.

### (4) Myth 4: naked mole‐rats are the only poikilothermic mammals

All thermoregulatory studies of naked mole‐rats agree that they are thermally labile, unable precisely to regulate body temperature (*T*_b_) when isolated from the colony (McNab, 1966; Withers & Jarvis, 1980; Buffenstein & Yahav, 1991; Goldman *et al*., 1999). The controversy associated with this ‘myth’ revolves around the use of the word ‘only’ and the Greek thermoregulatory terminology used to describe this highly variable *T*_b_. We agree that ‘only’ would be incorrect, as later thermoregulatory studies have revealed that other species of mole‐rats when isolated from their colonies also show pronounced thermolability (Bennett, Jarvis & Cotterili, 1993).

**Fig 2:**
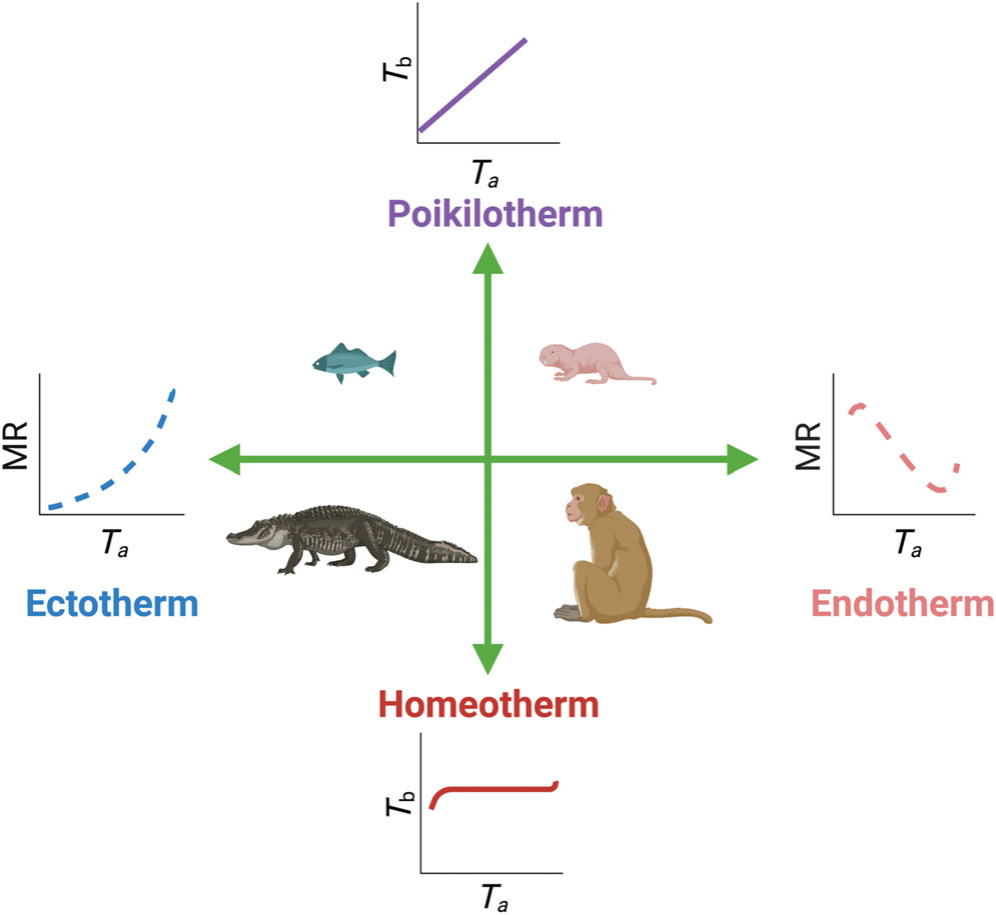
Thermoregulatory terms explained. Here, antonyms are shown opposite to each other. ‘Homeotherm’ refers to the maintenance of a core body temperature (*T*_b_) within a limited range (<2°C), whereas ‘poikilotherm’ refers to the thermal state whereby animals show large variability in *T*_b_ such that *T*_b_ is primarily dependent upon ambient temperature (*T*_a_). ‘Ectothermy’ and ‘endothermy’ refer to the two main mechanisms involved in heat acquisition. ‘Ectothermy’ relies mainly on behaviourally controlled heat exchange with the environment, whereas ‘endothermy’ refers to endogenous heat production. Using this cartoon, one can deduce that a crocodile, given its large thermal inertia, is able to maintain homeothermy using ectothermic mechanisms, i.e. shuttling from cooler to warmer areas to maintain body temperature, whereas a monkey primarily uses facultative thermogenesis to maintain a constant body temperature. By contrast, despite employing endothermy, given their large surface area to volume ratio and high rates of heat loss the naked mole‐rat cannot regulate *T*_b_ effectively outside of a very narrow range of environmental *T*_a_. MR, metabolic rate. Image modified from (Hislop & Buffenstein, 1994). Drawn with biorender.com

Buffenstein & Yahav (1991) considered the observed naked mole‐rat thermoregulatory profile of *T*_b_ closely tracking ambient temperature (*T*_a_) (when animals are housed individually in metabolic cages with dried incoming air) to be poikilothermic. The International Union of Physiological Sciences (IUPS) Thermal Commission define poikilothermy as the ‘large variability of *T*_b_ as a function of ambient conditions in organisms without *effective* autonomic temperature regulation’ (IUPS, 2001, p. 263). Braude *et al*. appear to consider the terms endothermy and poikilothermy as antonyms; however, the antonym of poikilothermy is actually homeothermy, a state where ‘*T*_b_ is maintained within a very narrow range’ (<2°C) (IUPS, 2001, p. 257) whereas endothermy refers to the endogenous mechanisms employed in adaptive heat generation for thermoregulatory purposes (see Fig. 2). Naked mole‐rats employ both the ectothermic mechanisms of huddling and basking (under heat lamps or on heating pads) and endothermic heat‐generating mechanisms, utilising their considerable depots of brown adipose tissue (BAT) (Daly, Williams & Buffenstein, 1997; Oiwa *et al*., 2020) for non‐shivering thermogenesis (Hislop & Buffenstein, 1994). As such it is undisputed that they employ endothermy. However, this mode of thermoregulation is not very *effective* in maintaining *T*_b_ of most animals when isolated from the colony, although it is generally adequate in larger breeding females (Urison & Buffenstein, 1994; Oiwa *et al*., 2020). Endothermy may be sufficient to regulate their low *T*_b_ (32–34°C) over the narrow range of *T*_a_s found in their hot, humid, equatorial burrows and under their hot, humid captive housing conditions. These environmental conditions reduce both the thermal gradient and vapour pressure gradient needed for heat loss. Group housing also reduces the surface area exposed for all avenues of heat exchange (Yahav & Buffenstein, 1991).

Braude *et al*. state that naked mole‐rats should rather be ‘considered endothermic and partially homeothermic, although with a limited ability to maintain a stable *T*_b_, but **not** poikilothermic’ (Braude *et al*., 2021, p. 380). In support of their premise that they are ‘partially homeothermic’, they mention that ‘skin temperature immediately upon capture was shown to vary mostly within the species thermoneutral zone (TNZ) despite a large temperature range inside the burrows (Holtze *et al*., 2018)’ (Braude *et al*., 2021, p. 380). This statement is hard to interpret: the TNZ refers to a range of *T*_a_s in which facultative thermogenesis is not employed, and not *T*_b_. Further, they mention that burrow temperatures are highly variable and imply that burrow *T*_a_s extend beyond the critical limits of the TNZ. Perhaps what the authors were meaning to say is that skin temperature immediately upon capture correlated with that of *T*_b_ within the TNZ. Given that warm mole‐rat burrows usually have a high relative humidity, thus reducing evaporative cooling at the skin–air interface, and the burrow–skin temperature gradient is also small reducing heat loss by other means (e.g. convection), it is highly likely that animals immediately upon capture would have a skin temperature that approximates core *T*_b_, and that it is less variable than if measured experimentally in metabolic chambers with fast‐flowing, dry air. However, this observation cannot be interpreted to mean these animals are ‘partially homeothermic’. Further compounding this interpretation is the fact that skin temperature (especially that of hands and feet) of most mammals is considerably more variable and generally lower than core *T*_b_.

Braude *et al*. emphasise that the differential between *T*_b_ and *T*_a_ varies among studies, ranging between ~1°C and 5°C (McNab, 1966; Withers & Jarvis, 1980; Buffenstein & Yahav, 1991). That this temperature differential differs among studies is in keeping with differences in experimental protocols; it can be influenced by (*i*) prior acclimation conditions, (*ii*) the provision and type of nesting material, (*iii*) the flow rate and dryness of air entering the metabolic chamber, (*iv*) the time in the metabolic chamber and if this is sufficient to allow acclimation, and (*v*) activity levels of the animals in question. For example, the larger differential observed by Withers & Jarvis (1980) reflects that in their study, animals were housed with considerable insulatory nesting material. Furthermore, when cooler temperatures were tested, the experimental duration was shortened, or animals were housed in groups and allowed to huddle, reducing the surface area for heat exchange. Nevertheless, all these studies report pronounced thermolability at *T*_a_ below the TNZ.

Further support of the use of the term ‘poikilothermy’ comes from studies in which animals were subjected to prolonged exposure (>1 year) to standard room temperature (23–25°C). Despite an increase in BAT and free thyroxine (T4), and a considerable time to acclimate to room temperature, the *T*_b_ (25–28°C) of these naked mole‐rats was more than 6°C below the *T*_b_ of naked mole‐rats when either caught in the wild (32–34°C) or housed at >27°C (Tb 32–35°C) in captivity (Buffenstein *et al*., 2001; Woodley & Buffenstein, 2002). Not only can naked mole‐rats tolerate prolonged periods with such a low *T*_b_, they can even successfully reproduce when their *T*_b_ is maintained at such low levels, although interbirth intervals, like that of poikilothermic viviparous reptiles, are extended and fewer and larger pups are born (Woodley & Buffenstein, 2001).

Collectively, the tolerance of a low *T*_b_, as well as their thermoregulatory profile shows that naked mole‐rats are indeed closer on the thermoregulatory spectrum to poikilotherms than to homeotherms (for an in‐depth review of this topic, see Willmer, Stone & Johnston, 2000). We do agree, however, that in their natural hot and humid milieu, living in large groups they would be able to regulate *T*_b_ better and could thus be regarded as stenotherms: animals able to regulate *T*_b_ over a **very** narrow range of *T*_a_s. The choice of terminology is clearly an area where there is a difference in opinion among scientists, but the possession of distinct poikilothermic traits is certainly not a ‘myth’.

### (5) Myth 5: naked mole‐rats have uniquely low thyroid hormone levels

Thyroid hormone is found in two principal forms: T4 (thyroxine) is the main circulating form, while T3 (triiodothyronine) is more biologically active and is synthesised from T4, by the enzyme iodothyronine deiodinase. Low free T4 levels have been reported in naked mole‐rats (Buffenstein *et al*., 2001; Buffenstein & Pinto, 2009) and Ansell's mole‐rat (Henning *et al*., 2014), although it is not known if this a shared trait in all mole‐rat species. To date, there are no peer‐reviewed published studies on T3 in naked mole‐rats. Rather, Braude *et al*. refer to a ‘news clip’ in *Science Daily* and use this source to state that naked mole‐rat T3 profiles are not low and that naked mole‐rats have a similarly low T4:T3 ratio to what they observe in *Fukomys anselli*. This news clip referred to a student interview concerning an upcoming conference poster presentation. That study included preliminary data, which upon repetition of the experiments could not be replicated – a finding attributed to problems encountered with the assay methods used. Given the lack of published data on T3 levels in naked mole‐rats, it is premature to conclude that they have a low T4:T3 ratio. Braude *et al*. go on to state that the resulting T4:T3 ratio, shared by naked mole‐rats and *F. anselli*, is ‘unique among mammals’ (Anderson, Nixon & Akasha, 1988), suggesting that the African mole‐rats ‘have a completely different thyroid hormone physiology’ (Braude *et al*., 2021, p. 380). The statement that either species has a ‘completely different thyroid hormone physiology’ is unfounded. Indeed, it remains to be proved that the T4:T3 ratio observed in captive *F. anselli* is even typical for Ansell's mole‐rat because, as evident in humans and other mammals, a diet deficient in iodine could explain a low T4:T3 ratio (Kabadi, 1982; Hulbert, 2000). Since iodine is an essential component of thyroid hormone synthesis, less T4 would be made and most of the T4 synthesised would then be converted into the active hormone T3. Measurements of thyroid stimulating hormone or experimental dietary interventions are needed to address this observation.

In summary, captive naked mole‐rats do have low thyroid hormone (T4) levels, but they are not unique, sharing this phenotype with *F. anselli*. However, there is no evidence to suggest that either species has a ‘completely different thyroid physiology’ to other mammals.

### (6) Myth 6: naked mole‐rat burrows are hypoxic and hypercapnic

The gaseous composition within an underground burrow system may vary considerably. This depends upon many abiotic and biotic factors, including soil depth, compaction and porosity, temperature, moisture, the number of burrow entrances, microorganisms within the soil, and the number of burrow inhabitants, to mention just a few (Buffenstein, 1996). A rarity among mammals, eusocial naked mole‐rats live in large family groups consisting of as many as 295 individuals (Brett, 1991). Notably, naked mole‐rats tend to collect in large numbers in a single basketball‐sized chamber (the nest), possibly to maintain social coherence, defend the matriarch, rest, share resources, and/or keep warm. This behaviour is routinely observed in captivity (Zions *et al*., 2020; Smith & Buffenstein, 2021). It is reasonable to theorise that so many animals respiring in a confined space would markedly alter underground atmospheric conditions, creating variable hypoxic and hypercapnic conditions (Brett, 1991). Unfortunately, there is very limited published data on the underground climate within the naked mole‐rat burrow system (Bennett, Jarvis & Davies, 1988; Holtze *et al*., 2018), primarily because precise measurements of air composition within burrows, and particularly nests, where large numbers of animals congregate for prolonged periods of time, have proved technically difficult to attain. For example, as outlined in Braude *et al*., McNab (1966) was unable to obtain accurate microclimate measurements within the naked mole‐rat burrow. Subsequent citations of McNab's work have presumed that the low O_2_ and high CO_2_ concentration reported therein for other predominantly solitary fossorial mammals are likely to be similar or exacerbated in eusocial naked mole‐rat burrows. A recent study by Holtze *et al*. (2018) reported only slight variation in ambient O_2_ and CO_2_ levels compared to above‐ground air sampled directly outside the burrow. However, these measurements were conducted in recently excavated burrows and may not reflect atmospheric conditions in older or deeper burrows, let alone fully occupied nests. Furthermore, these measurements were taken in December – the dry season in Ethiopia – when soils are more permeable to gases than when moist (Arieli & Nevo, 1991; Buffenstein, 1996) and may have resulted in less‐extreme conditions than those routinely encountered by naked mole‐rats in other seasons. Furthermore, according to table 1 of Holtze *et al*. (2018), the number of animals residing in the burrows during measurements ranged from 0 to 3 with a mean of 0.8 across 20 measurements, suggesting that many measurements were made in unoccupied portions of burrows. Absence of respiring individuals likely contributed to their inability to detect environmental hypoxia or hypercapnia. As such, their report may underestimate the true gaseous conditions in occupied naked mole‐rat nests.

Although there is sparse information regarding the gaseous composition of mole‐rat burrows in nature, a growing body of research has catalogued a remarkable array of adaptations to hypoxic and hypercapnic conditions in naked mole‐rats. Astoundingly, these animals can survive 80% CO_2_ for 5 h, and 18 min in 0% O_2_, more than 20 times longer than a mouse (Park *et al*., 2017). Adaptations supporting these capabilities encompass multiple aspects of physiology and biochemistry, suggesting that naked mole‐rats have evolved mechanisms to cope with environmental hypoxia and hypercapnia. For example, naked mole‐rats have gain‐of‐function mutations in their hypoxia‐inducible factor pathway (Kim *et al*., 2011), consistent with mutations in most other species adapted to hypoxia across millions of years (Pamenter *et al*., 2020). They also have smaller brain volumes (Orr *et al*., 2016), fewer neurons (Herculano‐Houzel *et al*., 2011), lower cardiac function (Grimes *et al*., 2014*b*) and lower basal metabolic rates (McNab, 1979), relative to body size and decrease oxygen requirements still further through cessation of thermogenesis (Kirby, Fairman & Pamenter, 2018), lowering heart rate (Pamenter *et al*., 2019) and metabolic rate (Pamenter, Dzal & Milsom, 2015). This ‘idling on low’ and ability to dial this down even further when acutely exposed to low O_2_ levels are characteristic of hypoxia‐tolerant species.

To survive elevated CO_2_ levels, naked mole‐rats counteract tissue acidosis (Park *et al*., 2017), show a lack of hypercapnia‐triggered increases in ventilation or behaviour (Clayson, Devereaux & Pamenter, 2020), have diminished substance P signalling under hypercapnia, and a decreased CO_2_ sensitivity of neuronal gap junctions (de Wolf, Cook & Dale, 2017). Correspondingly related to elevated CO_2_ levels, naked mole‐rats show a loss of sensitivity to noxious acidic stimuli *via* a mutation in the voltage‐gated sodium channel Na_V_1.7 (Smith *et al*., 2011) which is also found in many hibernating species (Liu *et al*., 2014), and a novel mutation in the gene encoding the neuronal potassium cotransporter KCC2 (Zions *et al*., 2020). This energy‐saving KCC2 variant makes naked mole‐rats dependent on higher CO_2_ levels to avoid seizures that would otherwise arise due to loss of brain inhibition.

Such remarkable tolerances to hypoxia and hypercapnia, and the multi‐faceted nature of the systemic adaptations that support them, suggest that these environmental stressors have applied evolutionary pressure to naked mole‐rats. While it is outdated to think that naked mole‐rats live in a steady state of hypoxia and hypercapnia, we do believe that the burrow/nest system means these animals likely experience bouts of hypoxia/hypercapnia interspersed with periods of relative normoxia/normocapnia. The statement in the subtitle above, then, likely represents an oversimplification rather than a ‘myth’.

## III. SENSORY ECOLOGY

### (1) Myth 7: naked mole‐rats are blind

Braude *et al*. concluded that the naked mole‐rat is not blind since they react to bright light flashes and summarised anatomical, physiological, and behavioural studies on naked mole‐rats spanning the past 18 years. These studies reveal an exceptionally thin optic nerve, an underdeveloped lateral geniculate nucleus, a visual cortex predominantly receiving input from tactile stimuli, a very disorganised retina and the prevalence of cataracts even in very young animals (Catania & Remple, 2002; Mills & Catania, 2004; Hetling *et al*., 2005; Crish, Dengler‐Crish & Catania, 2006; Xiao, Levitt & Buffenstein, 2006). While naked mole‐rats may be able to detect changes in luminance, they cannot form visual images (Nikitina *et al*., 2004; Hetling *et al*., 2005), and have no collicular or cortical response to visual stimuli (Catania & Remple, 2002). Although their poor vision would far exceed the criteria for ‘legal blindness’ in humans and they can accurately be considered functionally blind, we are not aware of any widespread ‘myth’ in the scientific literature that naked mole‐rats are literally blind, and unable to distinguish light and dark.

### (2) Myth 8: naked mole‐rats have degenerated hearing

The behavioural audiogram of the naked mole‐rat obtained by Heffner & Heffner (1993) shows a low‐frequency hearing limit of 65 Hz, a high‐frequency limit of 12.8 kHz [both at 60 dB sound pressure level (SPL)], and a lowest threshold of 35 dB SPL at 4 kHz. These observations were characterised in the title of that paper as ‘degenerate hearing and sound localization’. The controversy noted by Braude *et al*. centres on what is meant by ‘degenerate’. The Oxford English Dictionary (2021; online) defines this word as ‘Having lost the qualities proper to the race or kind; having declined from a higher to a lower type; hence, declined in character or qualities; debased, degraded’. Having ‘degenerate hearing’ would therefore mean that naked mole‐rats had poorer hearing than an ancestral species from which they evolved. In order to establish whether this is so, careful comparisons must be made.

**Fig 3:**
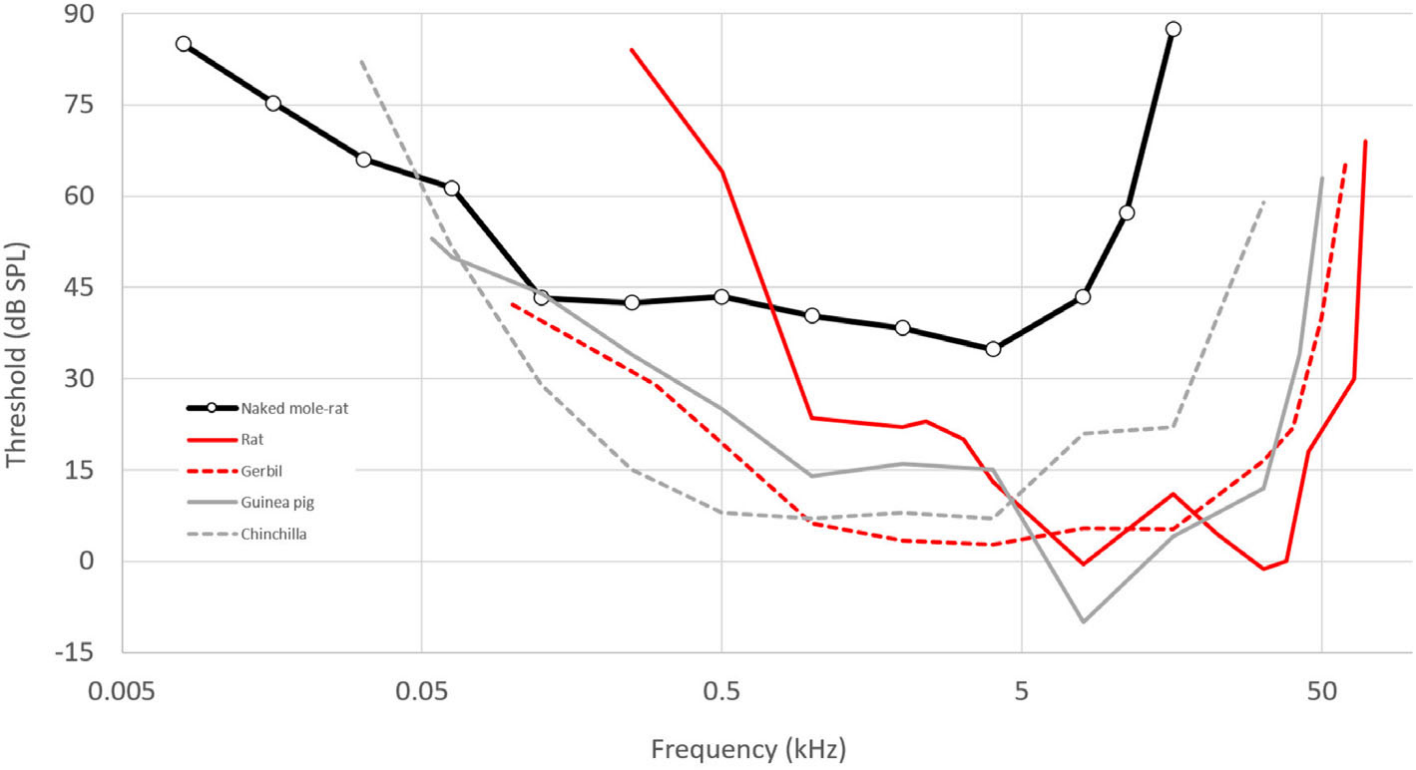
The behavioural audiogram of the naked mole‐rat in comparison with those of four other rodent species (see text for references). Rats and gerbils are murids; the other species are ctenohystricans.

Braude *et al*. compared the hearing of *Heterocephalus* with that of the rat. The hearing range of the hooded Norway rat (*Rattus norvegicus*) at 60 dB SPL extends from 530 Hz to 68 kHz, with lowest thresholds just below 0 dB SPL at 8 and 32 kHz (Heffner *et al*., 1994). The naked mole‐rat's thresholds are lower than those of the rat at frequencies of 500 Hz and below (Fig. 3), which might be interpreted as a potentially adaptive, low‐frequency shift in hearing range. Okanoya *et al*. (2018), on the other hand, used the gerbil (*Meriones unguiculatus*) instead of the rat for comparison. Gerbil hearing sensitivity at all frequencies tested (above 100 Hz) is better than that of the naked mole‐rat, and it extends to over 50 kHz at 60 dB SPL (Ryan, 1976). The gerbil's lowest threshold is 30 dB lower than that of the naked mole‐rat, at the same best frequency of 4 kHz. Its excellent low‐frequency hearing is associated with an expanded middle ear cavity (Ravicz & Rosowski, 1997), a feature commonly found in small mammals from arid regions (Mason, 2016*a*,*b*). The middle ear cavity of the naked mole‐rat, however, is much less voluminous (Mason, Cornwall & Smith, 2016).

The problem with both of the above comparisons, however, is that rats and gerbils come from a different rodent clade to *Heterocephalus*. Guinea pigs and chinchillas, which are much closer relatives, have excellent low‐frequency hearing (Fig. 3), more similar to the gerbil than the rat (Heffner, Heffner & Masterton, 1971; Heffner & Heffner, 1991). Both guinea pigs and chinchillas are substantially larger than naked mole‐rats; ideally, the hearing of the naked mole‐rat should be compared to that of a close relative (i.e. a ctenohystrican rodent) of more similar size. Compared to mammals in general, however, there is no doubt that the high‐frequency hearing limit is significantly lower than expected in the naked mole‐rat (Heffner, Koay & Heffner, 2001); the threshold at the best frequency is much increased and sound localisation abilities are poorer than in any surface‐dwelling mammal (Heffner & Heffner, 1993). This supports the claim that these aspects of hearing are indeed ‘degenerate’, according to the above definition.

Several aspects of naked mole‐rat ear morphology are indicative of reduced functionality and may contribute to these limitations (Mason *et al*., 2016). Recent work showed that these animals have missing or abnormally organised hair bundles on their cochlear outer hair cells (Pyott *et al*., 2020). The reduced hearing sensitivity of the naked mole‐rat may result from the lack of cochlear amplification, which was also demonstrated by Pyott *et al*. (2020).

Alterations in hearing arise evolutionarily from changes in selection. Reduced high‐frequency hearing and sound localisation could in principle arise from loss of selective pressure in the underground acoustic environment. Alternatively, these changes could come about through shifts in selective pressures. Evidence for this has been found at the gene and codon level, for hair bundle proteins involved in cochlear function (Pyott *et al*., 2020). In arguing that the hearing of naked mole‐rats is adapted to their environment, Braude *et al*. emphasise that their hearing range encompasses the airborne sound frequencies of a few hundred Hertz which propagate best in tunnels (Heth, Frankenberg & Nevo, 1986; Lange *et al*., 2007; Okanoya *et al*., 2018). The high thresholds even at these low frequencies have been explained by invoking the so‐called ‘stethoscope effect’, whereby certain frequencies may even be amplified (Lange *et al*., 2007). Based on the transmission of sound in pipes of similar diameter to naked mole‐rat tunnels, Okanoya *et al*. (2018) concluded that the mole‐rat should be able to hear conspecific calls over distances of some metres.

In conclusion, the hearing of the naked mole‐rat is indeed ‘degenerate’, in that several aspects of its audition have clearly deteriorated from what would reasonably be assumed to be the condition of its terrestrial ancestors. Hence, this is not a ‘myth’. That is not to say, however, that there are no selective pressures acting on its auditory system. Future studies, concentrating on comparisons with close relatives of *Heterocephalus*, are needed to establish whether the residual low‐frequency hearing of this animal has actually improved and, if so, whether this was driven by demands imposed by its underground niche, and/or the need for intraspecific communication.

### (3) Myth 9: naked mole‐rats are the most vocal rodents because they live in large groups

‘Most vocal’ could mean many things, but Braude *et al*. focus on the extent of the vocal repertoire. In the studies referenced in their paper (Pepper, Braude & Lacey, 1991; Knotkova *et al*., 2009; Bednářová *et al*., 2013; Vanden Hole *et al*., 2014; Dvořáková, Hrouzková & Šumbera, 2016), observational classification by the experimenter was the primary method for determining vocalisation types, making it difficult to standardise comparisons across species and to ensure that inter‐ and intra‐individual variability were appropriately weighted. Vocal complexity should not be strictly defined by the number of vocalisations within an animal's vocal repertoire: information content contained within each vocalisation must also be weighted (Freeberg, Dunbar & Ord, 2012). Recent work (Barker *et al*., 2021*b*), using supervised machine learning‐driven classification, demonstrated that complex information can be encoded in a single naked mole‐rat vocalisation, and Yosida & Okanoya (2009) demonstrated that when two animals meet head to head in a tunnel, social status affects the number of soft chirps emitted. These studies suggest that the vocal richness of the naked mole‐rat extends beyond their number of vocalisations to their ability to modify vocal cues to communicate multiple types of social information. We agree with Braude *et al*. that previous studies likely underestimated the number of vocalisations used by all members of the African mole‐rat family (summarised in Dvořáková *et al*., 2016). A recent comprehensive review (Barker *et al*., 2021*a*) identifies several additional naked mole‐rat vocalisations too, expanding the reported repertoire from 17 to 25. Taken together, the current data suggest that the naked mole‐rat is one of the most vocally complex rodent species, with a repertoire that even rivals that of primates (see McComb & Semple, 2005). We would not, however, seek to defend the contention that this rodent is necessarily the ‘most vocal’ since this is impossible to define; comparing counts of vocalisation types in a limited number of the more than 4000 species of rodents is specious.

The second part of ‘Myth 9’ refers to the driving force behind this vocal complexity. More robust comparative studies across the other African mole‐rats (Bathyergidae) are clearly needed before definitive conclusions about the interplay between social complexity (including group size) and vocal complexity can be made. As such, it is too soon to discount group size as an important contributor to vocal complexity within the Bathyergidae family, but group size is likely to be one of several key evolutionary drivers.

### (4) Myth 10: naked mole‐rats feel no pain

This is certainly a ‘myth’ circulating in the non‐scientific press, but this headline statement has not, it seems, been claimed in scientific articles. Indeed, perhaps the first comprehensive assessment of nociception in this species (Park *et al*., 2008), is entitled, ‘Selective inflammatory pain insensitivity in the African naked mole‐rat (*H. glaber*)’, the key word being ‘selective’. Normal nocifensive responses were reported for noxious heat and mechanical stimuli. Moreover, several detailed analyses from gene to behaviour have mechanistically explained the unusual nocifensive behaviour of this species. For example, Park *et al*. (2008) showed that naked mole‐rats show no nocifensive response to acid, which was later shown likely to result from a genetic variation in a gene encoding Na_V_1.7 (Smith *et al*., 2011). The gene variant in question renders the Na_V_1.7 channel more susceptible to inhibition by acid, thus shutting down action potential firing in nociceptor fibres. The same variant was found in the Cape mole‐rat (*Georychus capensis*) the only one of seven bathyergid species tested that was also insensitive to acid‐induced pain (Eigenbrod *et al*., 2019).

Braude *et al*. comment that, ‘contrary to Browe *et al*., 2020; Griffin, 2008, naked mole‐rats can sense pain *via* Aδ fibres, and respond normally to tissue damage (S. Braude, T.B. Hildebrandt & S. Holtze, personal observations; Browe *et al*., 2020; Griffin, 2008)’ (Braude *et al*., 2021, p. 382). This is misleading because Browe *et al*. (2020) do note that, ‘our behavioural data may reflect, to some extent, activity in neurons other than purinergic C‐fibers, namely A‐delta fibers’ (p. 11); moreover, the article from Griffin is a mini‐review that provides no discussion of fibre types, Aδ or otherwise. Furthermore, Park *et al*. (2008) provide a thorough electrophysiological characterisation of Aδ fibres in the naked mole‐rat compared to the mouse, finding a similar proportion of mechanoreceptors (D‐hairs) and Aδ‐mechanonociceptors, as well as a similar range of von Frey thresholds in both groups (see also Poulson *et al*., 2020). Thus, to our knowledge, there is no published literature arguing against a role for Aδ fibres in naked mole‐rat nociception. To clarify these issues better, we refer readers to a recent and extensive review of published data on naked mole‐rat nociception (Smith, Park & Lewin, 2020).

## IV. SOCIAL BEHAVIOUR AND REPRODUCTION

### (1) Myth 11: naked mole‐rats are the only eusocial mammals

This ‘myth’ encompasses two claims, firstly that naked mole‐rats are eusocial, and secondly that no other mammals are. There has been much controversy about the use of the term ‘eusocial’ in the mole‐rat literature. Originally, the term ‘eusocial’ was coined to include organisms that fitted three criteria: a reproductive division of labour, an overlap of generations, and cooperative care of the young (Michener, 1969). Two species of mole‐rats that were extensively studied in the 1980s appeared to meet these criteria, the naked mole‐rat and the Damaraland mole‐rat (Jarvis, 1981; Bennett & Jarvis, 1988). However, in the 1990s it became apparent that the criteria set to define eusociality in the social insects, including morphological castes, task specialisation for work‐related behaviours and even ‘overlapping generations’, did not necessarily apply to mole‐rats (Crespi & Yanega, 1995; Boomsma & Gawne, 2018). Lacking the formation of specialised castes prior to sexual maturity, naked mole‐rats would be excluded from their very stringent definitions (Crespi & Yanega, 1995). As a consequence, for vertebrate social species the assessment of skew in lifetime reproductive success of an individual was used to distinguish eusocial organisms from those that are social. Laboratory‐based studies revealed that fewer than 1% of individual naked mole‐rats were able to reproduce (Jarvis, 1991), whereas long‐term field studies on marked populations of Damaraland mole‐rats estimated that between 10 and 15% of individuals reproduced during their lifetime (Jarvis & Bennett, 1993). This is in marked contrast to the social insects – bees and termites – where a negligible number of individuals actually reproduce. These differences in reproductive skew were attributed to divergent developmental pathways, noting that complete metamorphosis in bees and ants allowed for greater specialisation for helping or breeding and hence higher levels of skew (Beekman, Peeters & O'Riain, 2006). In the 1990s and early 2000s, morphological and physiological castes were proposed for both breeding female naked mole‐rats and Damaraland mole‐rats and for non‐breeding Damaraland mole‐rats (O'Riain *et al*., 2000; Scantlebury *et al*., 2006; Young & Bennett, 2010). However, disagreements about the use of the terms ‘eusocial’, ‘caste’, ‘worker’ and ‘non‐worker’ have continued until today (see Zöttl *et al*., 2016; Thorley *et al*., 2018; Gilbert, Rossiter & Faulkes, 2020). While there may not be task specialisation in Damaraland mole‐rats, it does appear that animals change in the frequency of working behaviours with age (Thorley *et al*., 2018). Whether the naked mole‐rat can be regarded as the only ‘eusocial’ mammal therefore depends on the preferred definition of that term. Indeed, Jarvis & Bennett *et al*. (1993) have suggested that the Damaraland mole‐rat has also been considered eusocial. If eusociality is taken to encompass a multifactorial spectrum, naked mole‐rats would certainly be on it. Other social bathyergid species would be on that same spectrum too, although their smaller colony sizes and smaller skew in lifetime reproductive success would attest to a lower degree of eusociality.

### (2) Myth 12: colonies have castes of breeders and non‐breeders, involving frequent workers, infrequent workers, non‐workers, and dispersers

As pointed out by Braude *et al*., there is a distinct reproductive division of labour with some morphological specialisation in breeding queen naked mole‐rats (O'Riain *et al*., 2000), so it is puzzling that this forms part of the above ‘myth’. Queens become irreversibly morphologically distinct from female non‐breeders (O'Riain *et al*., 2000), but no such morphological distinction is apparent amongst the rest of the colony members, as discussed in both earlier (Jarvis, O'Riain & McDaid, 1991) and more recent research (Gilbert *et al*., 2020). However, the degree to which behavioural specialisation reflects subcastes among non‐breeding animals is still an area of active research and debate. The notion of frequent and infrequent worker castes mentioned in Jarvis' seminal paper (Jarvis, 1981) had already been questioned and modified as long ago as 1991, where evidence of age and body mass polyethisms were presented for some colonies (Faulkes & Abbott, 1991; Jarvis *et al*., 1991; Lacey & Sherman, 1991). All specialisations observed are behavioural and varied along a continuum, e.g. defence and work‐related activities (O'Riain & Jarvis, 1997; O'Riain & Faulkes, 2008). Recent, more extensive studies also report that tasks such as pup care behaviour, working behaviour, colony defence, and dispersal‐like behaviour are unevenly distributed among non‐breeding mole‐rats and somewhat stable among individuals across time (Mooney *et al*., 2015; Toor *et al*., 2020). Positive relationships between age and colony defence, and negative relationships between age and pup carrying, were also reported (Mooney *et al*., 2015). Gilbert *et al*. (2020) further highlight the complexity and variability in worker behaviour allocation. With regard to a ‘disperser caste’, distinctive disperser males have been shown to exist in laboratory colonies (O'Riain *et al*., 1996) and in the field (Braude, 2000). A recent follow‐up study on laboratory colonies replicated some components of the putative disperser subcaste, with a subset of larger/heavier non‐breeders showing motivation to leave the colony (Toor *et al*., 2020). Here, females were also noted to leave the colony in equal numbers, similar to what was reported in wild naked mole‐rats (Braude, 2000). It is still unclear to what extent naked mole‐rat subordinates show behavioural specialisation and if the increase in body fat that characterises the dispersive morph phenotype is simply a consequence of advancing age and preparing to disperse (Siegmann *et al*., 2021). These types of questions will only be resolved with considerably more field data examining life‐history traits and cooperative behaviours in their natural milieu. Moreover, it will be essential to determine the ontogeny and perpetuation of individual differences in the diverse behaviours of non‐breeders fully to answer the question of whether true non‐breeder sub‐castes can be considered to exist.

### (3) Myth 13: colonies have up to three male breeders (pashas)

The ‘myth’ regarding multi‐male mating by specific ‘breeders’ that form a long‐term bond with the queen is obscure. As Braude *et al*. correctly state, copulation itself does not guarantee paternity, and relatively few parentage studies utilising genetic markers have been conducted in naked mole‐rats. However, in a study examining two different litters within a colony, unequivocal genetic evidence revealed at least two males fathering the offspring within these litters, while a third putative breeding male could not be definitively excluded as a father (Faulkes *et al*., 1997). Clearly, there may be more than one breeding male in a colony, and generally the same few males are observed mating with the queen. However, there is no evidence to say that three is the upper limit, and hence this is not a ‘myth’.

### (4) Myth 14: colonies have a single queen

Evidence of dual queening has been reported many times; see Faulkes & Bennett (2009) for an extensive review on this topic. Jarvis *et al*. (1991) in the book *The Biology of the Naked Mole‐rat* provides percentages of single and dual queens in her large collection of naked mole‐rat colonies. However, in her 1980 collecting field trip in Kenya, all the colonies Jarvis trapped only had one breeding female. Similarly, over more than 25 years of fieldwork, only two instances of plural breeding were reported in 23 monitored colonies (Braude, 1991). In one of those colonies, the dual queens were both present a year later. The Buffenstein group has seen plural breeding occur seven times over a 10‐year period. All these instances of plural breeding occurred within only three out of 326 colonies (comprising 7542 individual mole‐rats; R. Buffenstein, personal observations). Similarly, the Holmes group has observed dual breeding in only two out of over 100 colonies (M. Holmes, personal observations). To date, in more than 40 years of maintaining naked mole‐rats in multiple laboratories, no one has reported any colony with three or more reproductive females. In all other social mole‐rats there are no multiple queens on record, but the colonies are much smaller (Bennett & Faulkes, 2000). Dual queening is clearly a relatively rare event both in captivity and in the wild, supporting the premise that most animals within a colony are reproductively suppressed. ‘Myth 14’ therefore represents an oversimplification rather than a complete falsehood.

### (5) Myth 15: not all females can become queens

Some individual females of any mammalian species will fail to breed successfully, so it must be trivially true that not all female naked mole‐rats can become queens. What this ‘myth’ really focuses on is whether there is a particular physiological condition which precludes breeding in some *Heterocephalus* females, which might serve some adaptive purpose. Beekman *et al*. (2006) classified all social vertebrates, including naked mole‐rats, as totipotent, i.e. having the potential at birth to become either a breeder or non‐breeder. Despite this, there is substantial variability in reproductive maturation of non‐breeding females. Some high‐ranking, non‐breeding females that are contenders for the queen position may activate while within the colony and usurp the queen by killing her. If subordinate females, 6 months of age or older, are removed from the colony and paired many, but not necessarily all, transition to breeders; some inevitably fail to reproduce. The Buffenstein, Holmes, Park and Smith groups commonly have a 50% pairing success if we randomly pair up individuals. Success can get closer to 100% if we carefully choose the females to remove from colonies for pairing. In this regard, we check if the female’ vagina becomes perforate when the queen is gravid, and screen for aggressive behaviour (Smith & Buffenstein, 2021). Some pairs successfully reproduce within three months after being paired. Given their long gestation, this implies that they come into oestrus within a week of being removed from the queen, but others take considerably longer (we have had a pair produce their first litter 2.5 years after pairing), and some pairs may never breed. Understanding the factors contributing to this variability is a focus of current research. Clearly, it could never be proved that *all* naked mole‐rat females could potentially become queens. Given our current knowledge, ‘not all females can become queens’ is not so much a ‘myth’ as a working hypothesis.

### (6) Myth 16: queens suppress workers with pheromones

This ‘myth’ is better described as a rejected hypothesis. As Braude *et al*. correctly point out, Jarvis (1981) in the concluding comments of her seminal paper suggests ‘pheromones may be involved in caste determination’ (p. 573). Given the wealth of literature on rodent pheromones at the time this paper was published, and the fact that naked mole‐rats use a communal toilet chamber, a role for urinary primer pheromones emitted by the queen in suppressing reproduction in the non‐breeders was a reasonable hypothesis to propose. Experimental investigation of the role of chemosignals in reproductive suppression in the late 1980s and early 1990s found no evidence for primer pheromones blocking reproduction, but instead implicated direct behavioural contact with the breeding queen (Faulkes & Abbott, 1993; Smith, Faulkes & Abbott, 1997). The Faulkes, Abbott & Jarvis (1990) paper referred to by Braude *et al*. to support the notion of social control of reproduction is not the correct citation in the context of whether or not pheromones are involved in reproductive suppression; this paper describes the physiology of suppression. With the exception of a BBC documentary (‘Supersense’) broadcast in 1989 that ignored the prevailing evidence and suggested a role for pheromones, pheromonal suppression of reproduction in naked mole‐rats has to our knowledge never been ‘mythologised’.

One recent paper of relevance to this discussion is that of Watarai *et al*. (2018). Faeces from the pregnant queen contained abundant oestrogen; non‐breeders fed these faeces increased their urinary oestrogen concentration, and their excreted faeces also contained abundant oestrogen. The authors proposed that faecal steroid consumption influences helping behaviour, which Braude *et al*. discount as a form of pheromonal transfer. However, faecal steroids fit the definition of pheromones in that they are transferred to conspecifics *via* the external environment, inducing a specific reaction. Transfer of oestrogen in this manner might in principle have a similar reproduction‐suppressing effect to its use in the combined contraceptive pill, although there is no direct evidence for this at present. Braude *et al*. state that ‘there is no evidence that naked mole‐rat non‐breeders ever consume queen faeces either in the wild or the laboratory setting’ (Braude *et al*., 2021, p. 384). However, both the queen and her subordinates practice coprophagy, eating faeces previously dropped by other colony members, and are commonly observed ‘begging’ for faeces from other members of their colony, which are then voided upon demand (Smith & Buffenstein, 2021). Although additional experimentation is needed, it is not impossible to believe that faeces of breeding females might be consumed by non‐breeders, and that this could potentially contribute to reproductive suppression.

### (7) Myth 17: queens shove workers to get them to work

Only one study proposed this (Reeve, 1992). While there are several citations of this paper to support the notion that subordinates in cooperative species are coerced into carrying out burrow maintenance and foraging activities, several subsequent studies have failed to support this idea (Jacobs & Jarvis, 1996; Clarke & Faulkes, 2001). Therefore, this bullying‐to‐work for the colony could be considered a ‘myth’.

### (8) Myth 18: naked mole‐rats never leave their natal colonies

This ‘myth’ was clearly debunked in the references Braude *et al*. cited in support of mole‐rats not being strictly subterranean (Myth 2). While most naked mole‐rats seldom leave the colony of their birth, dispersive morphs with distinct features have been reported (O'Riain *et al*., 1996) and dispersal has been discussed in some detail in a study on their ecology (Brett, 1991) as well as throughout the seminal book *The Biology of the Naked Mole‐rat* (Sherman, Jarvis & Alexander, 1991). Indeed, while direct evidence has yet to be acquired, all of the recent behavioural and genetic work supports the hypothesis that dispersal is an integral component of the ecology and evolution of this species (Braude, 2000; Ingram *et al*., 2015; Toor *et al*., 2020). Although more data are needed as to how and when dispersal occurs, and by which animals, there is no ‘myth’ in this regard.

### (9) Myth 19: naked mole‐rats are inbred

It is undisputed that naked mole‐rats are facultative inbreeders, a trait that sets them apart from other African mole‐rats. However, the initial evidence supporting extreme inbreeding has long been dismissed as more data, using more sophisticated techniques, have become available (Ingram *et al*., 2015; Chau *et al*., 2018). As Braude *et al*. point out, ‘consistent with Burda (1995), Clarke & Faulkes (1999) found that queens prefer to mate with unrelated males, and Ciszek (2000) demonstrated that naked mole‐rats avoid mating with siblings’ (Braude *et al*., 2021, p. 384). The Burda paper referred to in this quotation is only of indirect relevance as it does not refer to naked mole‐rats but rather to ‘common mole‐rats’, most likely a species of *Fukomys*.

## V. DEVELOPMENT, LONGEVITY, AGEING AND SENESCENCE

### (1) Myth 20: the GH/IGF axis is impaired in naked mole‐rats

In mammals, systemic insulin‐like growth factor (IGF1) levels are controlled by growth hormone (GH) as part of the somatotropic hormone axis. This hormone system regulates postnatal organismal growth, body composition and metabolism (Yakar, Werner & Rosen, 2018). Although direct functional characterisation of this ancestral hormone system in naked mole‐rats has not yet been investigated, the somatotropic endocrine axis undoubtedly operates in this species and there is a great deal of indirect evidence (see below) suggesting that it is downregulated. Mole‐rats have exceptionally slow growth rates both *in utero* and postnatally (Buffenstein *et al*., 2020). Their gestation is threefold longer than that of wild‐type mice and they exhibit postnatal somatic growth that continues for at least 18‐months (O'Riain & Jarvis, 1998). Naked mole‐rats share several other characteristics with GH/IGF1 mutant mice (Zhou *et al*., 1997). These include (*i*) their small size, relative to other sub‐Saharan mole‐rats (Bennett & Faulkes, 2000), (*ii*) sharply reduced rates of tumorigenesis (Junnila *et al*., 2013; Seluanov *et al*., 2018), (*iii*) increased insulin sensitivity (Kramer & Buffenstein, 2004; Junnila *et al*., 2013) coupled, remarkably, with high fat/lean mass ratios (O'Connor *et al*., 2002; Berryman *et al*., 2004), (*iv*) resistance of isolated fibroblasts to physical and chemical stressors (Salmon *et al*., 2005; Lewis *et al*., 2012), which likely stems from increased activity of nuclear factor erythroid 2‐related factor 2 (NRF2) in both dwarf mice and naked mole‐rats (Leiser & Miller, 2010; Lewis *et al*., 2015), (*v*) a circulating metabolomic signature reminiscent of fasting or hibernating mammals (Lewis, Rubinstein & Buffenstein, 2018), and (*vi*) slow age‐related declines of youthful phenotypes (Buffenstein, 2008; Edrey *et al*., 2012; Masternak *et al*., 2018; Buffenstein *et al*., 2020).

**Fig 4:**
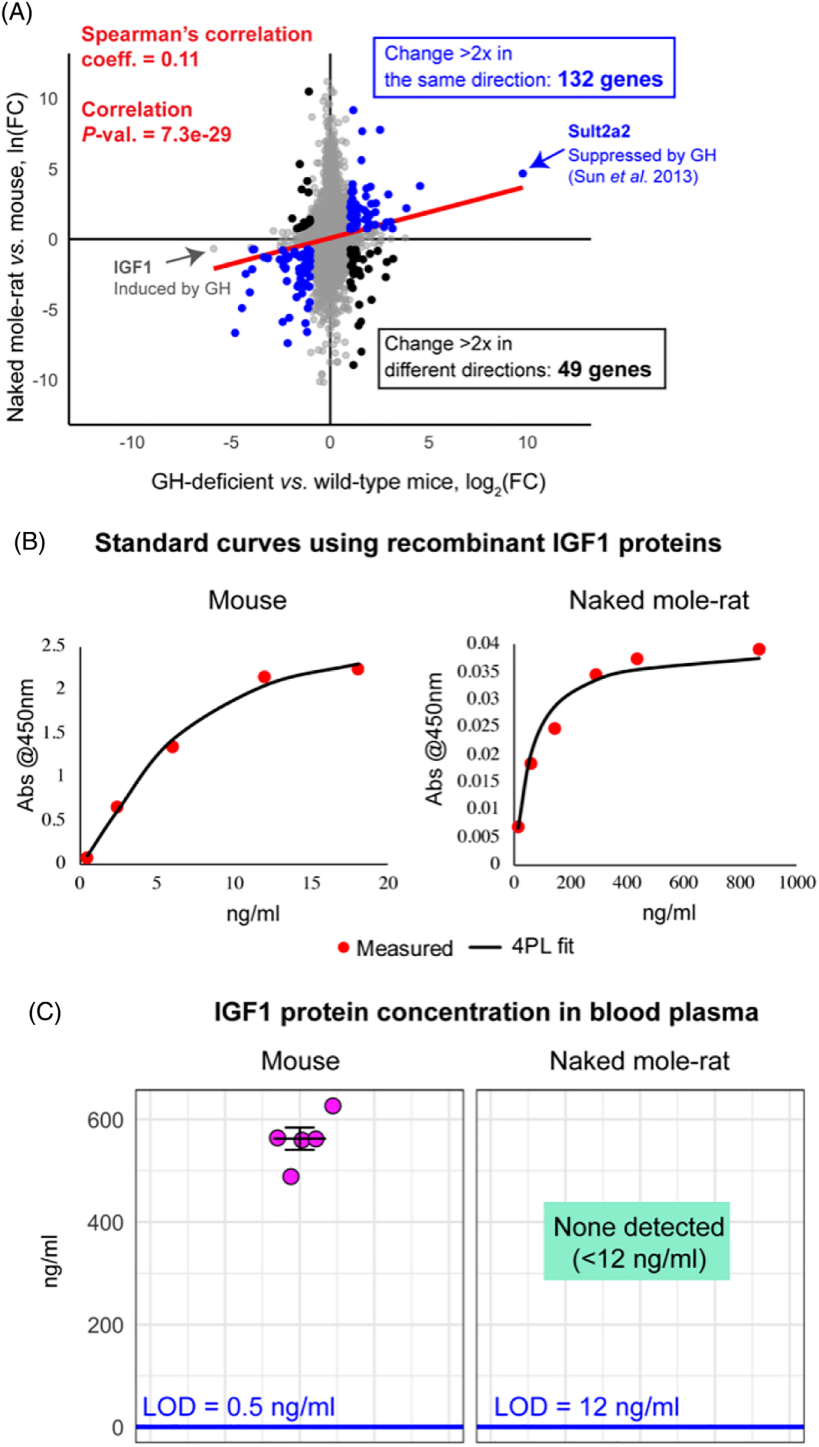
(A) Liver gene‐expression profiles of growth hormone (GH)‐deficient (*Ghrh* ^−/−^) mice and naked mole‐rats. Gene fold‐changes (FCs) were calculated relative to wild‐type mice for each organism, using ortholog conversion for naked mole‐rat genes (reciprocal best BLAST hits). Spearman's rank correlation coefficient for all genes on the plot and the correlation *P*‐value are shown in red. Of the genes that change substantially in both organisms (>twofold), those that change concordantly (blue) outnumber those that change non‐concordantly (black). Genes of special interest are labelled – insulin‐like growth factor 1 (*IGF1*; induced by GH) is down‐regulated in both organisms and *sulfotransferase family 2A* (*Sult2a2*) (repressed by GH) is upregulated in both organisms. Data for *Ghrh* ^−/−^ mice was obtained from GSE51108. Data for naked mole‐rats were generated by N. Rubinstein and R.B. and analysed by K.P. (B) Standard curves for commercial IGF1 enzyme‐linked immunosorbent assay (ELISA) (ALPCO 22‐IG1MS‐E01) generated using recombinant mouse and naked mole‐rat IGF1 proteins and four‐parameter logistic regression (4PL). (C) Concentration of IGF1 in blood plasma of young adult animals, measured using standard curves in B. Mice were 3.5 months old and naked mole‐rats were 2–2.3 years old; three females and two males were used in both groups. LOD, limit of detection.

There is also a transcriptional signature of GH deficiency in the naked mole‐rat liver. The Buffenstein and Kenyon groups found that transcriptomes of naked mole‐rats and dwarf mice resemble each other when comparing naked mole‐rat liver RNA sequencing (RNAseq) data to the published liver microarray data from GH‐deficient mice (Sun *et al*., 2013). There was a significant overall correlation between the two gene expression profiles (Fig. 4A; red line) and a significant overlap between the top differentially expressed genes (Fisher test *P*‐value = 4.7e−09, comparing top 5% of effect sizes). Gene set enrichment analysis (GSEA) indicates patterns of transcript expression in naked mole‐rats that are consistent with pathways that are perturbed in GH‐deficient mice, including upregulation of the tricarboxylic acid (TCA) cycle and respiratory electron transport, and down‐regulation of protein translation, mechanistic target of rapamycin (mTOR) signalling, and unfolded protein response.

Increased hepatic expression of IGF1 receptor (IGF1R) in mole‐rats (Fang *et al*., 2014) was proposed as a key argument against decreased IGF1 signalling in naked mole‐rats by Braude *et al*. However, IGF1R expression in the mammalian liver (regardless of species) is very low and variable (Wu *et al*., 2009; Uhlon, Fagerberg & Hallstrom, 2015). Indeed, an earlier naked mole‐rat study (Kim *et al*., 2011), reported *decreased* IGF1R levels in mole‐rats relative to mice, and in our own (Buffenstein and Kenyon groups) data we do not detect any IGF1R expression in the livers of either species. A more sensitive and widely accepted index of GH/IGF1 axis activity is the abundance of hepatic IGF1: the liver is the main source of circulating IGF1, as GH binding to the GH receptor on hepatocytes stimulates IGF1 transcription. Consistent with reduced activity of this hormone axis, we and others (Kim *et al*., 2011; Fang *et al*., 2014) report low levels of IGF1 messenger RNA (mRNA) in the naked mole‐rat liver (Fig. 4A). Circulating IGF1 protein levels of naked mole‐rats and mice were also measured using commercial enzyme‐linked immunosorbent assay (ELISA). To account for differences in antibody affinities to proteins from different species, separate standard curves using recombinant mouse or recombinant naked mole‐rat IGF1 protein were generated (Fig. 4B), and limits of IGF1 detection were calculated: 0.5 ng/ml for the mouse and 12 ng/ml for the naked mole‐rat protein. Average IGF1 concentration in mouse plasma was 560 ng/ml, consistent with published values, but no IGF1 protein was detected in naked mole‐rat plasma (Fig. 4C). While post‐translational modifications could account for lack of detection, these results are also consistent with previously published low circulating IGF1 levels (<12 ng/ml) in naked mole‐rats (Buffenstein, 2005).

Cultured naked mole‐rat fibroblasts secrete functional pregnancy associated plasma protein‐A (PAPP‐A) protease, and thereby can cleave IGF1 binding proteins and liberate IGF1 in local tissue microenvironments (Brohus *et al*., 2015). Therefore, it is unlikely that any differences in the activity of the GH/IGF1 axis between mice and naked mole‐rats result from differential regulation of IGF1 bioavailability. Instead, this likely arises upstream, at the level of GH production (Fig. 4). In addition, both Fang *et al*. (2014) and Brohus *et al*. (2015) reported that transcript levels of IGF2 are retained at high levels throughout adult life in naked mole‐rats (Kim *et al*., 2011; Brohus *et al*., 2015).

Although additional studies are needed to prove conclusively that the GH/IGF axis is ‘impaired’ in naked mole‐rats, current studies strongly suggest that this endocrine pathway is diminished in this species and that this ‘myth’ is likely to be true. This is an important and pertinent finding, as inhibition of GH/IGF1 signalling has been shown to extend lifespan in several other species (Kenyon, 2010).

### (2) Myth 21: naked mole‐rats are long‐lived because they have low oxidative stress and damage

The oxidative stress theory of ageing states that there is an imbalance between the production of reactive oxygen species (ROS) and their neutralisation by antioxidants, leading to an accumulation of oxidative damage with age that contributes to the physiological decline and loss of dynamic homeostasis during ageing (Harman, 2006). Braude *et al*. suggest that naked mole‐rat longevity does not align with the oxidative stress theory of ageing because naked mole‐rats paradoxically exhibit high levels of oxidative stress and damage, evident even at young ages (Andziak *et al*., 2006; Perez *et al*., 2009; Edrey *et al*., 2013). These high levels of damage are consistent with similar levels of ROS production observed in the heart mitochondria of mice and mole‐rats (Lambert *et al*., 2007; Munro *et al*., 2019), and equivocal findings regarding antioxidant defences that appear to be antioxidant, tissue and cell‐site specific (Munro *et al*., 2019; Andziak, O'Connor & Buffenstein, 2005; Viltard *et al*., 2019). Collectively, these findings appear to refute the concept that naked mole‐rats encounter low levels of oxidative stress.

However, the basic interpretation of the oxidative stress theory of ageing put forth by Braude *et al*. that ‘oxidative stress and damage inevitably result in premature ageing and age‐related diseases’ (Braude *et al*., 2021, p. 386) does not take into account the evolution of this theory over the 65 years since it was first proposed (Harman, 1956). For example, how oxidative damage accumulates over time may be more closely related to ageing than a static point comparison or comparisons between species. In this regard, naked mole‐rats align rather well with this ageing theory. In contrast to the steady rise in oxidative damage in mice as they age, while young naked mole‐rats exhibit higher levels of oxidative damage than seen in young mice, there is relatively little accumulation of oxidative damage in naked mole‐rat tissues throughout the life course (Andziak & Buffenstein, 2006; Perez *et al*., 2009).

In a similar regard, the molecular and cellular responses to oxidative stress/damage may prove more important to the regulation of ageing than the damage moieties themselves. Naked mole‐rat cells, in culture, are exceedingly resistant to multiple forms of stressors, including agents capable of generating oxidative stress (Salmon *et al*., 2008; Lewis *et al*., 2012). This is attributed to mechanisms in place that can repair or remove macromolecules or cells that have been irreversibly damaged. Numerous upregulated cytoprotective pathways, e.g. signalling from NRF2 and p53 (Lewis *et al*., 2010, 2015), upregulated DNA repair pathways (MacRae *et al*., 2015), and mechanisms involved in tolerating damage to proteins (Perez *et al*., 2009; De Waal *et al*., 2013) and lipids (Hulbert, Faulks & Buffenstein, 2006; Mitchell, Buffenstein & Hulbert, 2007), likely play a pivotal role in preventing age‐associated accrual of oxidative damage. These data collectively suggest that there is, in fact, some truth to this ‘myth’.

### (3) Myth 22: naked mole‐rat cells do not display cellular senescence

Cellular senescence is a protective mechanism to prevent the proliferation of cells that may be damaged or compromised by short telomeres. It has commonly been used as a sign of ageing and cancer resistance. Cellular senescence was first described by Leonard Hayflick in the 1960s, where he and colleagues observed that human cells undergo between 40 and 60 replications before they stop proliferating as a result of ‘replicative senescence’ (Hayflick, 1965). Several studies regarding telomere length and shortening have been reported in naked mole‐rats, with somewhat conflicting results. Multiple groups have reported that, compared to similarly sized but shorter‐lived mice, naked mole‐rats have relatively short telomeres and low levels of telomerase (Seluanov *et al*., 2007; Gomes *et al*., 2011). However, additional reports present evidence that naked mole‐rat telomeres do not shorten substantially with age, which they do in mice (Adwan Shekhidem *et al*., 2019; Leonida *et al*., 2020). More studies are needed to link these phenomena conclusively in the naked mole‐rat.

The measurement of actual senescence, both *in vitro* and *in vivo*, has proved to be extremely challenging in naked mole‐rats. Biochemical measures of senescence include β‐galactosidase staining, molecular markers like p16, and inflammatory senescence‐associated secretory phenotypes (SASPs). However, measurement of these markers and how they change with age or cellular replication in naked mole‐rats has proved problematic and remains largely unexplored. There is one study using β‐galactosidase staining, which showed that senescence plays a role in naked mole‐rat neonatal development, as well as RAS‐oncogene mediated and γ‐irradiation responses (Zhao *et al*., 2018). Additionally, Zhao *et al*. (2018) showed that after γ‐irradiation, SASP pathways are enriched in both naked mole‐rat and mouse skin fibroblasts suggesting that senescence can be induced in naked mole‐rat cells under certain conditions. Thus, given our current knowledge, we agree that Myth 22 is indeed a ‘myth’, or at least an oversimplification. However, more data are needed to assess the precise roles of senescence in replication, cancer, and ageing in the naked mole‐rat.

### (4) Myth 23: naked mole‐rats are immune to disease

Delaney *et al*. (2021) extensively catalogued diseases observed in captive naked mole‐rat colonies. Based upon fairly large numbers of *post‐mortem* analyses it is clear that, relative to other mammals, naked mole‐rats exhibit a low incidence of non‐infectious chronic diseases or age‐related degenerative diseases, such as osteoporosis, cardiovascular disease, neurodegeneration and cancer (Edrey *et al*., 2011; Buffenstein *et al*., 2012; Delaney *et al*., 2013, 2016*a*, 2021; Ward, Cartoceti & Delaney, 2020). Similarly, Braude *et al*. report a low incidence of non‐infectious and infectious diseases both from the literature and from a decade of observations at the Leibniz Zoological Gardens. However, incidence of specific diseases may vary among institutions: for example, chronic renal disease is rarely identified *post‐mortem* at the National Zoo colonies (Washington DC), but it is more prevalent in *post‐mortems* carried out at zoological gardens in Illinois (Delaney, Kinsel & Treuting, 2016*b*). This may reflect differences in husbandry, housing, or possibly genetic influences.

Adult individuals both in the wild (Hill *et al*., 1957) and in captivity also exhibit a very low incidence of infectious diseases (Delaney *et al*., 2021). However, an acute death syndrome has been anecdotally reported from multiple institutions where several animals within a particular colony that previously looked healthy are found dead with no signs of illness or lesions, suggestive of an acute contagious infection. When infections, specifically viral aetiologies, are found in captive colonies, these are often fatal. Both the Jarvis (University of Cape Town) and Sherman (University of Cornell) laboratories lost colonies to viruses, with the Jarvis group reporting that death was due to a coronavirus. This acute infection killed almost 50% of colony members (Ross‐Gillespie, O'Riain & Keller, 2007). Moreover, experimental infection with a recombinant herpes simplex virus type 1, which is avirulent to mice, caused 100% mortality when administered to naked mole‐rats (Artwohl *et al*., 2009). These findings are further supported by studies that their immune cell populations lack natural killer cells, the cells responsible for immune surveillance and eradication of virally infected cells (Hilton *et al*., 2019). In conclusion, although naked mole‐rats do appear relatively resistant to many types of chronic disease, it is clearly a ‘myth’ that they are fully immune to disease. This is not a contention that we are aware of in the scientific literature, however.

### (5) Myth 24: naked mole‐rats do not get tumours or cancer

In the first large‐scale report on lack of cancer prevalence, it was not claimed that naked mole‐rats ‘do not get tumors or cancer’, but rather that, ‘our unusual mortality pattern may reflect the low susceptibility of these animals to cancer’ (Buffenstein, 2008) (p. 441). Over time we will acquire a more accurate assessment of the actual degree of cancer resistance. In a comparison of incidence with other wild rodents, the cancer rates in wild animals are difficult to measure accurately and cancer prevalence in wild‐caught house mice is influenced by environmental factors (Gardner *et al*., 1973; Harper, Leathers & Austad, 2006). However, naked mole‐rats appear highly resistant compared to other laboratory rodents. By studying them, significant insights into tumorigenesis, mechanisms of resistance, prevention and treatment strategies may arise. It will also be important to elucidate in future studies how naked mole‐rat individuals respond to several types of experimental carcinogenesis induction.

### (6) Myth 25: naked mole‐rats have extremely large hyaluronan

Hyaluronan is ubiquitously found in the extracellular matrix of cells and most tissues throughout the bodies of mammals (Fraser, Laurent & Laurent, 1997). Hyaluronan increases the hydration and viscoelastic properties of the surrounding extracellular matrices and spaces due to its high net‐negative charge (Engstrom‐Laurent, 1997). Abundant high molecular weight hyaluronan was found both in the culture media conditioned by naked mole‐rat fibroblasts and in naked mole‐rat tissues (Tian *et al*., 2013; Taguchi *et al*., 2020; Takasugi *et al*., 2020). Hyaluronan was detected by multiple methods including Alcian blue staining, including all necessary controls such as treatment with specific hyaluronan‐degrading enzymes (Tian *et al*., 2013; Taguchi *et al*., 2020; Takasugi *et al*., 2020). Thus, we reject the criticism by Braude *et al*. that Alcian blue staining performed in this way has no specificity.

Hyaluronan size is determined by the balance between synthesis and degradation. Braude *et al*. argue that it is unclear how the two mutations in the conserved catalytic core of the naked mole‐rat hyaluronan synthase 2 (HAS2) enzymes could lead to higher molecular weight, as a variety of sizes was produced when naked mole‐rat HAS2 was expressed in cancer cells. This is not surprising at all, as different cancer cell lines differ greatly in expression levels of the hyaluronan‐degrading enzymes, hyaluronidases.

How high is ‘high’ molecular weight? It was originally observed that naked mole‐rat hyaluronan had a very high molecular weight (6–12 MDa) (Tian *et al*., 2013). However, determining the exact size of large hyaluronan molecules is complicated being potentially affected by the isolation procedure. The largest available hyaluronan size markers typically end at 6 MDa. A recent analysis has demonstrated that naked mole‐rat hyaluronan has a high average molecular weight, but not greater than 2.5 MDa, whether from tissue or cell supernatant (Del Marmol *et al*., 2021).

What is important is that hyaluronan in the naked mole‐rat may contribute to the unique physiology of this species (Gorbunova, Takasugi & Seluanov, 2020; Takasugi *et al*., 2020). Indeed, it has been shown using atomic force microscopy that hyaluronan extracted from naked mole‐rat tissues forms a range of assemblies (corresponding to a distribution of molecular weights), including supercoiled structures that are absent from hyaluronan extracted from mouse tissue, as well as an ability spontaneously to form gels. These properties likely contribute to the elasticity of naked mole‐rat skin and may have the potential to function as a barrier to tumour invasion (Kulaberoglu *et al*., 2019).

Tian *et al*. (2013) reported the first evidence that hyaluronan contributes to cancer resistance in the naked mole‐rat. In regard to this resistance of naked mole‐rat cells to oncogenic transformation, Braude *et al*. state that ‘a recent study that failed to reproduce these findings’ (Braude *et al*., 2021, p. 388), but fail to cite the reply to Hadi *et al*. (2020) published in the same issue, which demonstrated that the discrepant observations between Tian *et al*. (2013) and Hadi *et al*. (2020) originate from the different expression levels of oncogenic HRas (HRas G12V). When oncogenic Harvey Rat sarcoma virus (HRas) is driven by a long terminal repeat (LTR) promoter to express at a moderate level, Tian *et al*. (2013) and Liang *et al*. (2010) independently reported that naked mole‐rat fibroblasts are more resistant to oncogenic transformation than mouse cells. However, when HRas G12V is expressed at a higher level, naked mole‐rat cells generate tumours in xenografts (Zhao *et al*., 2020). The original studies did not claim that naked mole‐rat cells can never be transformed by oncogenes, but rather, as is stated in the title of the paper by Liang *et al*. (2010), they are more resistant to transformation than those of mice. Overall, given the susceptibility of naked mole‐rat cells to transformation, it is important to identify the mechanisms responsible for naked mole‐rat tumour resistance, including any potential role for their hyaluronan in creating a cancer‐resistant tissue microenvironment.

### (7) Myth 26: naked mole‐rat cells have early contact inhibition that prevents cancer

Primary cultures of dermal fibroblasts from adult naked mole‐rats are relatively difficult to establish under the standard conditions routinely used for humans and mice, and these cells grow more slowly than those of embryos or neonates. The growth of naked mole‐rat fibroblasts is particularly sensitive to culture conditions: if conditions are suboptimal, the proliferation of these cells slows, or stops completely. We have found that even ostensibly minor changes in our cell culture maintenance protocols are sufficient to impact proliferation. Similarly, unlike the remaining viable fibroblasts of mice which may continue to proliferate even when most of the mouse fibroblasts are killed following toxin exposure, naked mole‐rat fibroblasts readily stop proliferating when subject to toxin exposure without any obvious signs of senescence (Lewis *et al*., 2012). This stress‐induced growth arrest likely reflects an important role in the cancer resistance of the naked mole‐rat.

Some authors have observed that naked mole‐rat fibroblasts secrete abundant hyaluronan into the culture medium which increases its viscosity and arrests cell proliferation before cells reach confluence *via* cluster of differentiation 44 (CD44) receptor signalling (Seluanov *et al*., 2009; Tian *et al*., 2013). It takes 4–5 days for sufficient hyaluronan to accumulate to trigger cell growth arrest. Frequent medium changes remove this hyaluronan and result in confluent cell culture with naked mole‐rat cells attaining higher densities than observed for mouse cells, suggesting that contact inhibition is not a cell autonomous process (Liang *et al*., 2010; Lewis *et al*., 2012; Narayan *et al*., 2021). Similarly, some authors have observed that degradation of hyaluronan by bacterial hyaluronidase generates a confluent cell culture (Tian *et al*., 2013), whereas others have observed no change in cell confluence when hyaluronidase is added to cell cultures (Del Marmol *et al*., 2021). Considering the abundant hyaluronan present in the microenvironment of naked mole‐rat tissues, the potential role of hyaluronan signalling in naked mole‐rat cell culture may contribute to the cancer resistance of this species.

The differences in culture conditions for naked mole‐rat cells used by different laboratories resulted in lively debate. However, attempts have been made to understand why the data are at variance (Seluanov *et al*., 2009; Liang *et al*., 2010; Lewis *et al*., 2012; Narayan *et al*., 2021). Having built upon the knowledge gleaned from these divergent findings, we now have a far better understanding of how conditions affect naked mole‐rat cell growth and susceptibility to cell cycle arrest. Braude *et al*. refer to unpublished data from their own group (T. B. Hildebrandt, N. Kichler, S. Holtze & M. Vyssokikh, in preparation) to dismiss this ‘myth’. Without any relevant information on their growth conditions, it is impossible to evaluate whether their statement contradicts or supports the model of naked mole‐rat growth described in this section.

### (8) Myth 27: naked mole‐rats are non‐ageing

As adult organisms age, their risk of dying increases exponentially. This concept of age‐dependent mortality, first proposed by the actuarial scientist Benjamin Gompertz, is widely regarded as a fundamental law of mortality (Gompertz, 1825) and a prominent metric in the comparative biology of ageing (Sacher, 1977; Kirkwood, 2015). The Gompertz–Makeham laws provide a context for observing the rate at which a species ages, measured in terms of the doubling rate of mortal hazard across a population (therefore referred to as ‘demographic’ ageing). Ruby *et al*. (2018) analysed a large compendium of lifespan data (>3200 animals) in order to estimate the rate of demographic ageing for naked mole‐rats and found that the risk of dying did not increase at all with age, even at ages more than 25 times the age of sexual maturity. Dammann *et al*. (2019) previously wrote a ‘comment’ to that publication, expressing concern about the potential of missing data from the distant past to confound the conclusion. An additional new analysis that specifically addressed the concerns of Dammann *et al*. (2019) was undertaken using left‐censorship as specified by Kaplan & Meier (1958) for the analyses of incomplete demographic data (Ruby, Smith & Buffenstein, 2019). That new analysis yielded the *same* conclusion as the original paper: a lack of demographic ageing in naked mole‐rats across decades of life (Ruby *et al*., 2019).

Furthermore, we disagree that these findings are undermined by earlier incidence reports of reduced activity or other physiological or morphological changes in some older animals (Edrey *et al*., 2011; Lewis & Buffenstein, 2016; Delaney *et al*., 2021). For example, these studies have reported that skin colour becomes more uniform and paler in older subordinates as well as in breeders at any age. While this may be akin to hair greying without associated pathology, there may be other ecophysiological explanations for why this occurs. The paper ‘Successful ageing and sustained good health in the naked mole‐rat’, reports on the observed signs of physiological declines occurring wherever these are evident, even if these incidences, like that of cancer, are rare (Edrey *et al*., 2011). However, this and other studies also report that the great majority of animals remain active, continue to breed well into their third decade, show no menopause, unchanged cardiac function, body composition and metabolic profiles and indeed retain numerous paedomorphic traits well into adulthood (O'Connor *et al*., 2002; Buffenstein, 2008; Edrey *et al*., 2012; Grimes *et al*., 2014*a*; Lewis & Buffenstein, 2016; Buffenstein *et al*., 2020; Buffenstein & Craft, 2021). The health deteriorations that we have observed in naked mole‐rats (e.g. cataracts and osteoarthritis) appear to change neither in severity nor frequency as age increases: for example, cataracts have been observed in juveniles (<3 months) and adults in every decade of life (Edrey *et al*., 2011; M.A. Hanes & R. Buffenstein, personal communication). From an examination of data covering the first three decades of life, death and disease both appear to be stochastic and independent of chronological age, consistently supporting the classification of the naked mole‐rat, at least until their third decade of life, as a ‘non‐ageing’ mammal (Ruby *et al*., 2018). As such, based upon Gompertzian laws as well as age‐related phenotypes, that naked mole‐rats are non‐ageing mammals is not a ‘myth’.

## VI. TAXONOMY

### (1) Myth 28: naked mole‐rats are the single member of a taxonomic family

Braude *et al*. state ‘The taxonomic status of *Heterocephalus* is not so much a myth as a scientific error’ (Braude *et al*., 2021, p. 389), referring to the contention by Patterson & Upham (2014) that the differences between *Heterocephalus* and the other bathyergids warrant its placement within its own family, Heterocephalidae. Braude *et al*. dispute some of the evidence used by Patterson & Upham (2014), and argue that *Heterocephalus* should be retained in the Bathyergidae. A recent review of the phylogeny and biogeography of the Bathyergidae reveals that the species richness of the Bathyergidae needs to be revisited in a systematic and comprehensive manner (Visser, Bennett & van Vuuren, 2019). We therefore feel that it is premature to regard *Heterocephalus* as a member of a separate family to the Bathyergidae. However, while we acknowledge that the placement of familial designations is to some extent subjective, we dispute the contention that the taxonomic proposal of Patterson & Upham (2014) represents either a ‘myth’ or a ‘scientific error’. Biosystematic revisions, based on differing combinations of characteristics (e.g. genes, fossil records, and morphological traits), as well as emerging sophisticated analytical techniques, lead to competing and evolving hypotheses that should converge towards the true solution over time.

## VII. CONCLUSIONS

(1) We would not deny that there is, and will continue to be, misinformation about the naked mole‐rat circulating in the popular press and electronic media. However, we feel that many of the ‘myths’ identified by Braude *et al*. (2021) serve to create misunderstanding where little exists. We hope to have demonstrated herein that these do not, in fact, represent widespread and unsubstantiated beliefs among the scientific community (see Table 1 for a summary). We have identified several common themes which unite several of these ostensible ‘myths’: (*i*) some ‘myths’, as presented in headline form, are better described as oversimplifications. For example, we agree that the naked mole‐rat is not completely hairless, nor completely blind, and it is likely not to be continuously exposed to hypoxic and hypercapnic conditions (myths 1, 6, 7) – but there is an element of truth to all of these statements. (*ii*) Other so‐called ‘myths’ may or may not have an element of truth, but we simply do not have enough information at the present time to be sure (e.g. myths 5, 9, 15, 28). Those myth‐statements could represent the ‘premature conclusions’ referred to by Braude *et al*., but it is not clear that anyone in the field believes that these cases are, indeed, closed. (*iii*) Alleged ‘myths’ based on conclusions of earlier studies which have subsequently been revised in line with new evidence (e.g. myths 16, 17, 19, 21). (*iv*) Aspects of naked mole‐rat biology that are currently interpreted differently by different research groups, perhaps because of semantic disagreements (e.g. myths 4, 8, 11, 12). Many of the 28 ‘myths’ combine one or more of these common themes.

(2) The addition of exclusive terms such as ‘strictly’, ‘uniquely’, ‘the only’, ‘the most’ or ‘never’ contributes to some of the other myth‐statements being false or at least questionable, sometimes because of similarities between naked mole‐rats and other sub‐Saharan mole‐rats. Braude *et al*. have helped to bring prominence to studies of other African mole‐rats, which is important in serving to put some of the naked mole‐rat data into a wider context, but similarities with close relatives do not negate the interest value and importance of the naked mole‐rat as a model species.

(3) Unfortunately, it is inevitable that researchers will find and cite some papers while missing others, so there will always be some perpetuation of outdated or incorrect ideas. This is the case in any scientific field, of course. Competing hypotheses and interpretations are also inevitable, and indeed are a healthy part of the scientific endeavour. Fortunately, in the fullness of time, science is self‐correcting – as Thomas Kuhn (quoting Francis Bacon) stated, ‘Truth emerges more readily from error than from confusion’ (Kuhn, 1962, p. 38). Although we respectfully disagree with many of the contentions of Braude *et al*. (2021), and we have attempted to show why, we encourage debate and welcome challenging articles that serve to drive reconsideration and discussion. We do, however, reject their labelling of evidence‐based hypotheses, even if outdated, as ‘myths’, and we feel that this is particularly inappropriate when it comes to competing theories where there is disagreement amongst scientists. We hope that readers of the Braude *et al*. paper have not been misled into thinking that naked mole‐rat research involves any more confusion and error than any other active area of research in biology, and that research in this burgeoning field would be wasted effort. To the contrary, we firmly believe that there is much to be learnt from this non‐conventional laboratory species.

(4) Decades of research by many groups across the world, with very different areas of expertise, has contributed to a great deal now being known about the naked mole‐rat. Some of this is proving to be directly pertinent to understanding and mitigating human disease. This knowledge base will continue to be refined and built upon. No doubt, some of our current understanding will need to be revised in the light of evidence yet to be uncovered, and although some of the current debates will be settled, new ones will surely arise. Every new publication helps us better to understand the unusual biology of this curious little mammal.
